# Meta-analysis of the Age-Dependent Efficacy of Multiple Sclerosis Treatments

**DOI:** 10.3389/fneur.2017.00577

**Published:** 2017-11-10

**Authors:** Ann Marie Weideman, Marco Aurelio Tapia-Maltos, Kory Johnson, Mark Greenwood, Bibiana Bielekova

**Affiliations:** ^1^Neuroimmunological Diseases Unit, National Institute of Neurological Disorders and Stroke, National Institutes of Health, Bethesda, MD, United States; ^2^PECEM, Facultad de Medicina, Universidad Nacional Autónoma de México, Mexico City, Mexico; ^3^Bioinformatics Section, National Institute of Neurological Disorders and Stroke, National Institutes of Health, Bethesda, MD, United States; ^4^Department of Mathematical Sciences, Montana State University, Bozeman, MT, United States

**Keywords:** clinical trials, neuroimmunology, neuroinflammation, clinical practice, meta-analysis

## Abstract

**Objective:**

To perform a meta-analysis of randomized, blinded, multiple sclerosis (MS) clinical trials, to test the hypothesis that efficacy of immunomodulatory disease-modifying therapies (DMTs) on MS disability progression is strongly dependent on age.

**Methods:**

We performed a literature search with pre-defined criteria and extracted relevant features from 38 clinical trials that assessed efficacy of DMTs on disability progression. We fit a linear regression, weighted for trial sample size, and duration, to examine the hypothesis that age has a defining effect on the therapeutic efficacy of immunomodulatory DMTs.

**Results:**

More than 28,000 MS subjects participating in trials of 13 categories of immunomodulatory drugs are included in the meta-analysis. The efficacy of immunomodulatory DMTs on MS disability strongly decreased with advancing age (*R*^2^ = 0.6757, *p* = 6.39e−09). Inclusion of baseline EDSS did not significantly improve the model. The regression predicts zero efficacy beyond approximately age 53 years. The comparative efficacy rank derived from the regression residuals differentiates high- and low-efficacy drugs. High-efficacy drugs outperform low-efficacy drugs in inhibiting MS disability only for patients younger than 40.5 years.

**Conclusion:**

The meta-analysis supports the notion that progressive MS is simply a later stage of the MS disease process and that age is an essential modifier of a drug efficacy. Higher efficacy treatments exert their benefit over lower efficacy treatments only during early stages of MS, and, after age 53, the model suggests that there is no predicted benefit to receiving immunomodulatory DMTs for the *average* MS patient.

## Introduction

With the expansion of the multiple sclerosis (MS) drug armamentarium, it is becoming exceedingly difficult to make informed decisions regarding their comparative efficacy. Experts debate whether first line MS therapy should consist of low- versus high-efficacy drugs and at what age, if any, it is appropriate to withdraw immunomodulatory disease-modifying therapies (DMTs).

This debate is confounded by the widely accepted classification of MS patients into relapsing-remitting (RRMS), secondary-progressive (SPMS), and primary-progressive MS (PPMS) subtypes. While these phenotypical categories prove useful in clinical trial designs and conceptual thinking about MS, they *de facto* dichotomize the continuous process of MS evolution. Indeed, a patient with MS does not go to sleep one day with RRMS and wake up next day with SPMS. Instead, there is a period, often lasting several patient-years, in which a clinician encounters considerable uncertainty in differentiating RRMS from SPMS. An analogous uncertainty is frequently encountered in differentiating SPMS from PPMS based on ambiguity in recollecting event(s) in a patient’s history which may or may not represent MS relapses.

More importantly, the justification for categorizing MS patients in drug development and clinical care was mechanistically rooted in the long-held belief that the amount of intrathecal inflammation is vastly greater in RRMS than in progressive MS [especially PPMS (1)] patients. This explained the lack of efficacy of immunomodulatory DMTs in progressive MS and justified the exclusion of patients with progressive MS from RRMS trials, irrespective of whether they fulfilled the remaining inclusion criteria. However, this belief was recently disproven by objective data: on one hand, patients with all three MS subtypes were shown to have identical levels of central nervous system (CNS) T- and B-cell-mediated inflammation (2), and, on the other hand, immunomodulation by the B-cell depleting therapy ocrelizumab inhibited disability progression in PPMS (3).

Instead, the alternative hypothesis for the relative lack of efficacy of immunomodulatory drugs in progressive MS resides in two mutually non-exclusive, *continuous* processes: compartmentalized, terminally differentiated intrathecal inflammation (2), and/or neurodegeneration (4). Compartmentalization of inflammation can be defined as the establishment of a permissive environment for long-term survival and *in situ* activation of the non-resident immune cells, mediated by the formation of tertiary lymphoid follicles in the CNS tissue (5–7). Compartmentalization is a continuous process that starts at MS onset and evolves over time, which means that it is predominantly (but not exclusively) seen in older subjects with progressive MS (2, 5–7). Compartmentalized inflammation is inaccessible to orally or intravenously administered MS drugs with poor CNS penetrance (8, 9). Additionally, chronic inflammation induces a parallel process called terminal differentiation of immune cells. This causes antigen-specific lymphocytes derived from the cerebrospinal fluid of patients with longstanding MS to proliferate less when compared with T cells derived from MS patients with short disease duration (10). Differentiation is again a continuous process through which naïve cells, which secrete only interleukin-2, but proliferate rapidly, evolve through repeated cycles of antigen-driven activation to cells that produce many different cytokines, but have limited proliferative capacity (10). This may explain the relative lack of efficacy of small-molecule cytostatic agents that target cells in the proliferation cycle in patients with progressive MS.

Alternatively, the immunomodulatory DMTs have relatively low-efficacy in progressive MS because inflammation, although present, may not be the most important driver of disability progression. Indeed, neurodegenerative mechanisms such as mitochondrial dysfunction (11), oxidative damage (12), hypoxia (13–15), endoplasmic-reticulum stress (16), and astroglial toxicity (17) have been identified predominantly (but not exclusively) in progressive MS. Even as it remains unknown which of these (if any) contribute to disability progression, it is rational to assume that these too are continuous, rather than dichotomized processes.

Thus, together with homogeneity of MS susceptibility alleles among phenotypical MS subgroups (18) and epidemiological data that demonstrate that MS patients from all three phenotypical categories achieve major disability milestones at a similar age (19), the aforementioned data support the unifying concept that PPMS and SPMS are biologically indistinguishable and represent the evolved/later stage of the MS disease process (2, 20).

If this concept is true, then the loss of therapeutic efficacy of immunomodulatory DMTs in MS should also be a continuous, rather than dichotomous process. Thus, one can hypothesize that the efficacy of immunomodulatory drugs will negatively correlate with MS duration. Because the exact onset of MS is unknown for most MS subjects, we can restate the hypothesis that efficacy of MS DMTs will negatively correlate with patient age.

Consequently, the primary goal of this project was to perform a meta-analysis of all blinded, randomized clinical trials of immunomodulatory DMTs that reported disability risk reductions to test the null hypothesis that drug efficacy is independent of age. If we can model efficacy of current DMTs as a continuum, we may better predict the benefit of immunomodulatory DMTs for individual patients. Thus, the secondary goal was to develop age-adjusted objective model(s) to compare the efficacy of MS drugs. The resulting optimized models can be used in future research studies (such as those that seek to identify prognostic biomarkers or biological processes that underlie MS severity) to adjust measured data for the efficacy of administered therapies.

## Materials and Methods

### Selection of Trials

**Figure 1:**
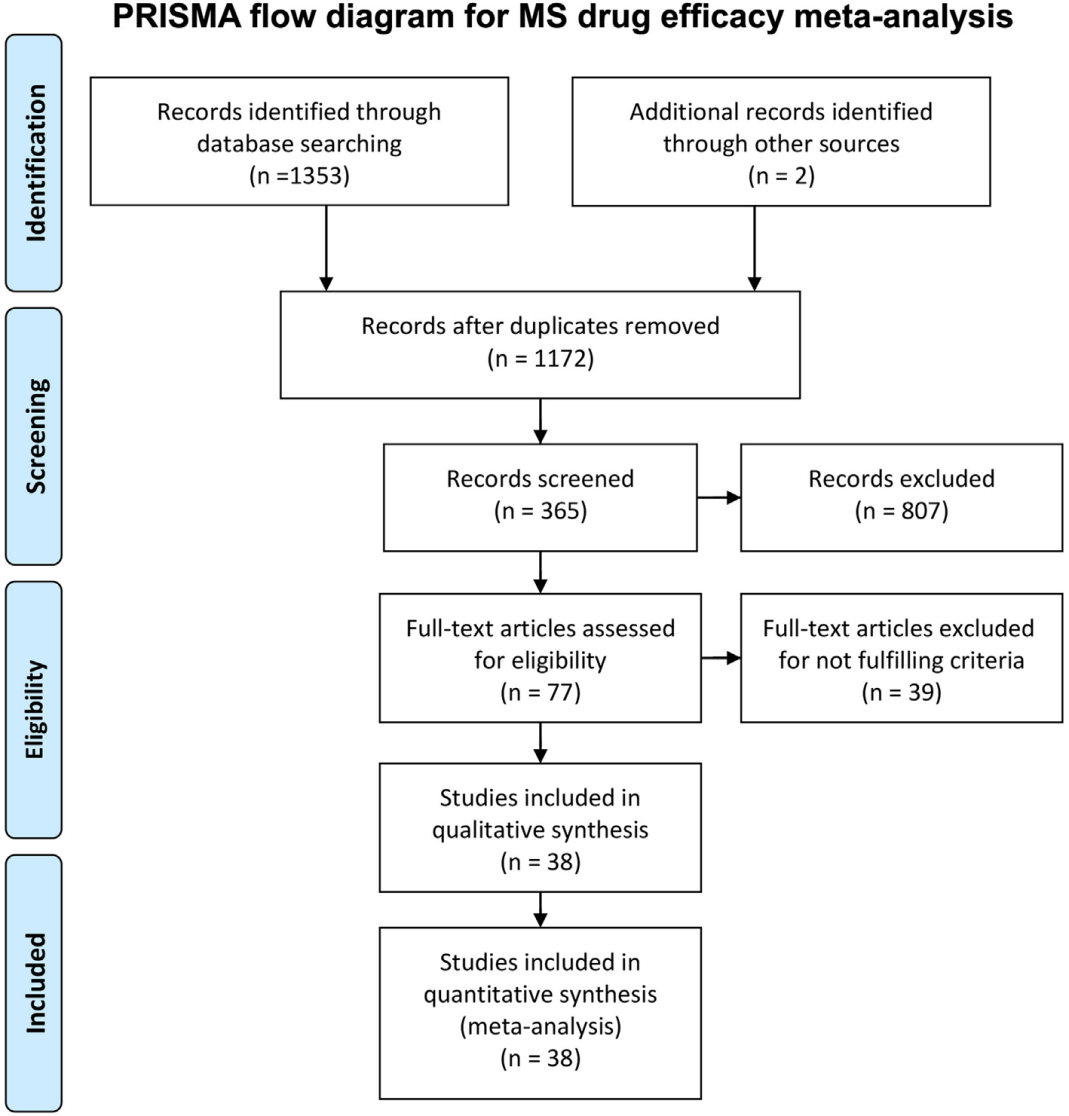
PRISMA flow chart for immunomodulatory multiple sclerosis drug efficacy meta-analysis. The diagram summarizes our search strategy for including clinical trials in the meta-analysis.

We conducted a systematic review of immunomodulatory DMTs for MS. The PubMed search filter “clinical trial” and the key words “multiple sclerosis,” in combination with “interferon” (*n* = 842), “glatiramer acetate” (*n* = 192), “fingolimod” (*n* = 67), “dimethyl fumarate” (*n* = 18), “teriflunomide” (*n* = 15), “mitoxantrone” (*n* = 52), “daclizumab” (*n* = 29), “natalizumab” (*n* = 86), “alemtuzumab” (*n* = 23), “rituximab” (*n* = 15), “ocrelizumab” (*n* = 3), “laquinimod” (*n* = 10), and “siponimod” (*n* = 1) were used for screening relevant studies. In addition, we searched the public domain (including Clinicaltrials.gov) for non-PubMed sources with complete efficacy data on drugs currently under development. The inclusion criteria for selecting the trials were (1) randomized clinical trial in any MS subtype, (2) double-blinded or rater-blinded trial, (3) trial duration of at least a year (when counted in weeks, at least 48 weeks), (4) comparison between drug and placebo or between a drug and an active comparator (interferon beta), (5) the proportion of patients with confirmed disability progression (CDP; a change in EDSS confirmed in a subsequent follow-up visit after 3 or 6 months) measured in both groups as an outcome in the study, and (6) in trials with two arms, at least one arm of the trial used the FDA-approved dose of the drug (this arm was chosen for the analyses). The PRISMA flowchart (21) (Figure 1) provides details regarding the disposition of screened studies.

The following information was extracted from each study: author, trial name, year, drug, dose, control group (placebo or active comparator), MS subtype, sample sizes, trial duration, baseline patient characteristics, CDP in each group by the end of the trial, and *p*-values (Table 1, also see Supplementary Material for an Excel spreadsheet containing all trial data and accompanying calculations). For trials that did not list a hazard ratio, we calculated the percent inhibition of disability progression (%IDP) as follows:

(1) $$\%\text{IDP}=\left(1-\frac{{\hat{p}}_{\text{drug}}}{{\hat{p}}_{\text{placebo}}}\right)\times 100\%$$

where ${\hat{p}}_{\text{drug}}$ represents the proportion of patients from the drug group with CDP, and ${\hat{p}}_{\mathrm{placebo}}$ represents the proportion of patients from the comparator group with CDP by the end of the trial. This formula is equivalent to a relative risk reduction, where the ratio between ${\hat{p}}_{\mathrm{drug}}$ and ${\hat{p}}_{\mathrm{placebo}}$ represents the relative risk of disability progression.

**Table 1:** Clinical trials used for weighted regression analysis.

Index	Trial	Year	Experimental arm	Control arm	*n*	Trial duration (years)	Weight	Age at baseline (years)	IDP (%)		*p*-Value
									Raw	Recalculated	
A1	CAMMS223	2008	Alemtuzumab	Interferon beta-1a (Rebif)	223	3.00	386.25	32.35	58.00	73.69	0.005
A2	CARE-MS I	2012	Alemtuzumab	Interferon beta-1a (Rebif)	563	2.00	796.20	33.07	30.00	55.12	0.220
A3	CARE-MS II	2012	Alemtuzumab	Interferon beta-1a (Rebif)	628	2.00	888.13	35.12	42.00	60.39	0.008
D1	SELECT	2013	Daclizumab HYP	Placebo	397	1.00	397.00	35.94	57.00	57.00	0.021
D2	DECIDE	2015	Daclizumab HYP	Interferon beta-1a (Avonex)	1841	2.76	3,059.41	36.30	16.00	40.63	0.160
DF1	CONFIRM	2012	Dimethyl fumarate	Placebo	541	1.84	733.39	37.50	21.00	21.00	0.250
DF2	DEFINE	2012	Dimethyl fumarate	Placebo	817	1.84	1,108.56	38.30	38.00	38.00	0.005
F1	FREEDOMS	2010	Fingolimod	Placebo	843	2.00	1,192.18	36.90	30.00	30.00	0.020
F2	FREEDOMS II	2014	Fingolimod	Placebo	713	1.97	1,001.06	40.35	17.00	17.00	0.227
F3	INFORMS	2016	Fingolimod	Placebo	823	4.32	1,709.60	48.50	11.99	11.99	0.217
F4	TRANSFORMS	2010	Fingolimod	Interferon beta-1a (Avonex)	860	1.00	860.00	36.35	25.32	47.14	0.250
G1	Copolymer 1 MS Study Group	1995	Glatiramer acetate	Placebo	251	2.00	354.97	34.45	12.20	12.20	NS
G2	PROMiSe	2007	Glatiramer acetate	Placebo	935	3.00	1,619.47	50.40	13.00	13.00	0.175
G3	REGARD	2008	Glatiramer acetate	Interferon beta-1a (Rebif)	764	1.84	1,036.65	36.75	25.64	46.76	0.117
G4	BEYOND	2009	Glatiramer acetate	Interferon beta-1b (Betaseron)	1343	2.00	1,899.29	35.60	4.76	34.04	0.680
G5	CONFIRM	2012	Glatiramer acetate	Placebo	532	1.84	721.18	37.34	7.00	7.00	0.700
G6	CombiRx	2013	Glatiramer acetate	Interferon beta-1a (Avonex)	487	3.00	843.51	38.31	−14.92	14.08	Not given
I1	MSCRG	1996	Interferon beta-1a (Avonex)	Placebo	301	2.00	425.68	36.80	37.25	37.25	0.020
I2	Montalban et al.	2009	Interferon beta-1b (Betaseron)	Placebo	73	2.00	103.24	48.70	20.83	20.83	0.314
I3	Leary SM et al.	2003	Interferon beta-1a (Avonex)	Placebo	35	2.00	49.50	44.50	−20.00	−20.00	NS
I4	PRISMS	1998	Interferon beta-1a (Rebif)	Placebo	283	2.00	399.52	34.90	17.76	17.76	<0.05
I5	PRISMS	1998	Interferon beta-1a (Rebif)	Placebo	278	2.00	392.44	34.90	28.73	28.73	<0.05
I6	SPECTRIMS	2001	Interferon beta-1a (Rebif)	Placebo	259	3.00	447.74	42.97	12.00	12.00	0.305
I7	SPECTRIMS	2001	Interferon beta-1a (Rebif)	Placebo	248	3.00	428.68	42.63	17.00	17.00	0.146
I8	INFB MS Study Group	1995	Interferon beta-1b (Betaseron)	Placebo	244	5.00	545.60	35.50	23.21	23.21	0.096
I9	EUSPMS	1998	Interferon beta-1b (Betaseron)	Placebo	718	2.75	1,190.67	41.00	21.73	21.73	0.005
I10	NASPMS	2004	Interferon beta-1b (Betaseron)	Placebo	625	3.00	1,082.53	46.80	5.59	5.59	0.590
I11	ADVANCE	2014	Pegylated interferon beta-1a (Plegridy)	Placebo	1012	0.92	970.96	36.60	38.00	38.00	0.038
I12	BRAVO	2014	Interferon beta-1a (Avonex)	Placebo	672	2.00	950.35	38.00	26.00	26.00	0.130
L1	ALLEGRO	2012	Laquinimod	Placebo	1106	2.00	1,564.12	38.70	36.00	36.00	0.010
L2	BRAVO	2014	Laquinimod	Placebo	659	2.00	931.97	37.10	31.00	31.00	0.063
M1	MIMS	2002	Mitoxantrone	Placebo	124	2.00	175.36	39.98	63.64	63.64	0.036
N1	AFFIRM	2006	Natalizumab	Placebo	942	2.30	1,429.04	36.00	54.00	54.00	<0.001
N2	SENTINEL	2006	Natalizumab + IFN-beta-1a (Avonex)	IFN-beta-1a (Avonex) + Placebo	1171	2.30	1,776.44	38.90	24.00	24.00	0.020
N3	ASCEND	2016^a^	Natalizumab	Placebo	887	1.84	1,203.54	47.20	−6.67	−6.67	0.753
O1	OPERA I	2016	Ocrelizumab	Interferon beta-1a (Rebif)	821	1.84	1,113.99	37.00	43.00	58.90	0.010
O2	OPERA II	2016	Ocrelizumab	Interferon beta-1a (Rebif)	835	1.84	1,132.99	37.30	37.00	54.19	0.020
O3	ORATORIO	2016	Ocrelizumab	Placebo	731	2.85	1,234.07	44.60	25.00	25.00	0.040
R1	OLYMPUS	2009	Rituximab	Placebo	439	1.84	595.67	49.90	23.00	23.00	0.144
S1	EXPAND	2016^a^	Siponimod	Placebo	1363	1.75	1,803.08	48.00	21.00	21.00	0.013
T1	TEMSO	2011	Teriflunomide	Placebo	547	2.07	786.51	37.73	23.70	23.70	0.080
T2	TEMSO	2011	Teriflunomide	Placebo	540	2.07	776.44	38.00	29.80	29.80	0.030
T3	TOWER	2014	Teriflunomide	Placebo	601	1.54	746.87	37.63	5.00	5.00	0.762
T4	TOWER	2014	Teriflunomide	Placebo	564	1.60	714.14	38.17	32.00	32.00	0.044

*Of the 44 clinical trials analyzed, 6 of these trials were conducted using two treatments arms and one control arm to give 38 independent trials. For the weighted regressions, each arm was considered as an independent trial, and patients in the control arms were divided equally between the treatment arms. Age at baseline was calculated by adding the products of mean age and sample size for each group (experimental and control) and then dividing by the total sample size. The raw value for percent inhibition of disability progression (%IDP) was calculated using Eq. 1 in Section “Materials and Methods” and then recalculated (for trials with interferon beta as active comparator) using Eq. 7. The *p*-value denotes the significance of the reported difference in confirmed disability progression between trials arms. For trials that did not report a *p*-value (due to non-significant results), these *p*-values are denoted by NS. A complete table with trial references, doses, and calculations can be found in the uploaded Excel file in Supplementary Material*.

*^a^Unpublished Trial*.

### Weighting Scheme

Based on validated methodology used in previous meta-analyses (22, 23), we calculated a weight for each trial using the following formula:

(2) $$\mathrm{Weight}=n\sqrt{D}$$

where *n* is the trial sample size and *D* is the trial duration in years, thus, assigning a larger weight to trials with a larger sample size and a longer duration. For trials with multiple arms, the patients in the placebo group were divided equally between the treatment arms. For example, the CONFIRM 2012 trial intention-to-treat population consisted of twice-daily dimethyl fumarate (*n* = 359) and glatiramer acetate (*n* = 350) (24). The placebo group (*n* = 363) was divided equally between the two experimental groups, so that the sample size used for modeling was 359 + 363/2 (rounded) and 350 + 363/2 (rounded), respectively. By using this methodology, we prevented false inflation of weights associated with double-counting patients in trials of multiple analyzable arms.

### Weighted Regression for Interferon Beta versus Placebo

To estimate a drug’s efficacy against placebo in trials that used interferon beta as an active comparator, we performed a weighted regression of all interferon-beta trials as a function of age and used the mean age at baseline to calculate ${\text{IDP}}_{\text{IFN}-\beta \text{versus}\text{Placebo}}$ for each trial. All analyses were conducted in statistical software R v3.3.1 (RStudio v1.0) (25, 26), and the accompanying R scripts can be found in Supplementary Material. By Eq. (1), it holds true that,

(3) $${\text{IDP}}_{\text{Drug versus Placebo}}=100\%\left(1-\frac{{\hat{p}}_{\text{Drug}}}{{\hat{p}}_{\text{Placebo}}}\right)=100\%\left(1-\frac{{\hat{p}}_{\text{Drug}}}{{\hat{p}}_{\text{IFN}-\beta }}\cdot \frac{{\hat{p}}_{\text{IF}N-\beta }}{{\hat{p}}_{\text{Placebo}}}\right).$$

By rearranging Eq. 1 to solve for the respective proportions, it also holds true that,

(4) $$\frac{{\hat{p}}_{\text{Drug}}}{{\hat{p}}_{\text{IFN}-\beta }}=1-\frac{{\text{IDP}}_{\text{Drug}\;\text{versus}\;\text{IFN}-\beta }}{100}$$

and

(5) $$\frac{{\hat{p}}_{\text{IFN}-\beta }}{{\hat{p}}_{\text{Placebo}}}=1-\frac{{\text{IDP}}_{\text{IFN}-\beta \;\text{versus}\;\;\text{Placebo}}}{100}$$

so that, by substitution, the adjusted IDP as a percentage can be calculated by,

(6) $$\begin{array}{l} {\text{IDP}}_{\text{Drug}\;\text{versus}\;\text{Placebo}}=100\%\left(1-\left(1-\frac{{\text{IDP}}_{\text{Drug}\;\;\text{versus}\;\;\text{IFN}-\beta }}{100}\right)\cdot \left(1-\frac{{\text{IDP}}_{\text{IFN}-\beta \;\text{versus}\;\;\text{Placebo}}}{100}\right)\right). \end{array}$$

Alternatively, this equation can be thought of as a percent change between trials by considering how much a patient would have progressed while on placebo versus interferon beta compared with interferon beta versus another drug such as alemtuzumab. For example, consider a patient of baseline age 32 who progresses by 0.1 EDSS points per year while on placebo and 0.063 EDSS points per year while on interferon beta (assuming interferon beta has a 37% efficacy at that age). Assume then that alemtuzumab prevents disability progression by 58% when compared with interferon beta. Then, this same patient would progress by only 42% of 0.063 EDSS points or approximately 0.026 EDSS points per year. The percent change between alemtuzumab versus placebo is then (0.1 − 0.026)/0.1 × 100% or 74% IDP, which matches the answer received by using Eq. 6 with ${\text{IDP}}_{\text{Drug}\;\text{versus}\;\text{IFN}-\beta }=58\%$ and ${\text{IDP}}_{\text{IFN}-\beta \;\text{versus}\;\;\text{Placebo}}=37\%$.

### Simple Weighted Regression and Dichotomization of Treatments

To compare efficacies of DMTs, we fit the following simple weighted regression to all drug trials:

(7) $${\text{IDP}}_{W_{i}}=\beta_{0W}+\beta_{1W}{\text{Age}}_{i}+\varepsilon_{W_{i}}$$

where the subscripts $W_{i}$ are indices of the weighted terms/parameters and ε is the error term. We treated this regression as the response of the *average* patient prescribed an *average* DMT. We then computed the weighted standardized residuals (27) ($\varepsilon_{W_{i}}$) for all trials and repeated only for trials of FDA-approved DMTs in approved indications, as follows:

(8) $$\varepsilon_{W_{i}}=\frac{\sqrt{W_{i}}\left({\text{IDP}}_{i}-{\hat{\text{IDP}}}_{W_{i}}\right)}{\mathrm{SD}\left({\text{IDP}}_{i}-{\hat{\text{IDP}}}_{W_{i}}\right)}$$

where ${\text{IDP}}_{i}-{\hat{\text{IDP}}}_{W_{i}}$ is the difference between the observed and fitted values (indicated with a hat), and the denominator is the standard deviation (SD) of the residuals. The latter was used to compute weighted residual means for each drug; high-efficacy drugs had weighted residual means above the regression line and low-efficacy drugs had weighted residual means below the regression line.

### Weighted Regression with Interaction Term

Following the residual analysis, a step-down testing procedure was employed by starting with a model that included Age, Efficacy of FDA-approved DMTs in approved indications (indicator coded 0 for low and 1 for high), and EDSS and ending with a model that included only interactions between Age and Efficacy (terms involving EDSS were not retained in the model as discussed below), as follows:

(9) IDPWi=β0W+β1W(Agei)+β2W(Efficacyi)+ β3W(Agei)(Efficacyi)+εWi

where the subscripts $W_{i}$ are indices of the weighted terms, and ε is the error term. This results in a single model that can be used to predict the IDP as dependent on low- or high-efficacy treatment and the age at which this treatment is administered. The step-down testing justified exploring the two groups separately, and we used the dichotomization of FDA-approved DMTs into low- and high-efficacy categories to fit two separate models. Residual diagnostics were checked and no evidence of violation of model assumptions was suggested.

### Difference in Means: Low- versus High-Efficacy Therapy

We used a *t*-test to determine that the age adjustments should be different between the low- and high-efficacy drugs. To determine the point at which there is no difference between low- and high-efficacy treatments, we estimated the difference between the mean low- and high-efficacy %IDP as a function of age and plotted the 95% confidence interval (CI) for this difference to determine the age at which this difference approached zero.

## Results

### Age Alone Explains a Large Proportion of Variance in the IDP

Thirty-eight trials (Table 1, Supplementary Material) matched our inclusion/exclusion criteria (Figure 1) and were used for the subsequent analysis. We computed the weights for each clinical trial in the meta-analysis dependent on the number of patients enrolled and trial duration as described (see Eq. 2 in Materials and Methods) (22, 23).

**Figure 2:**
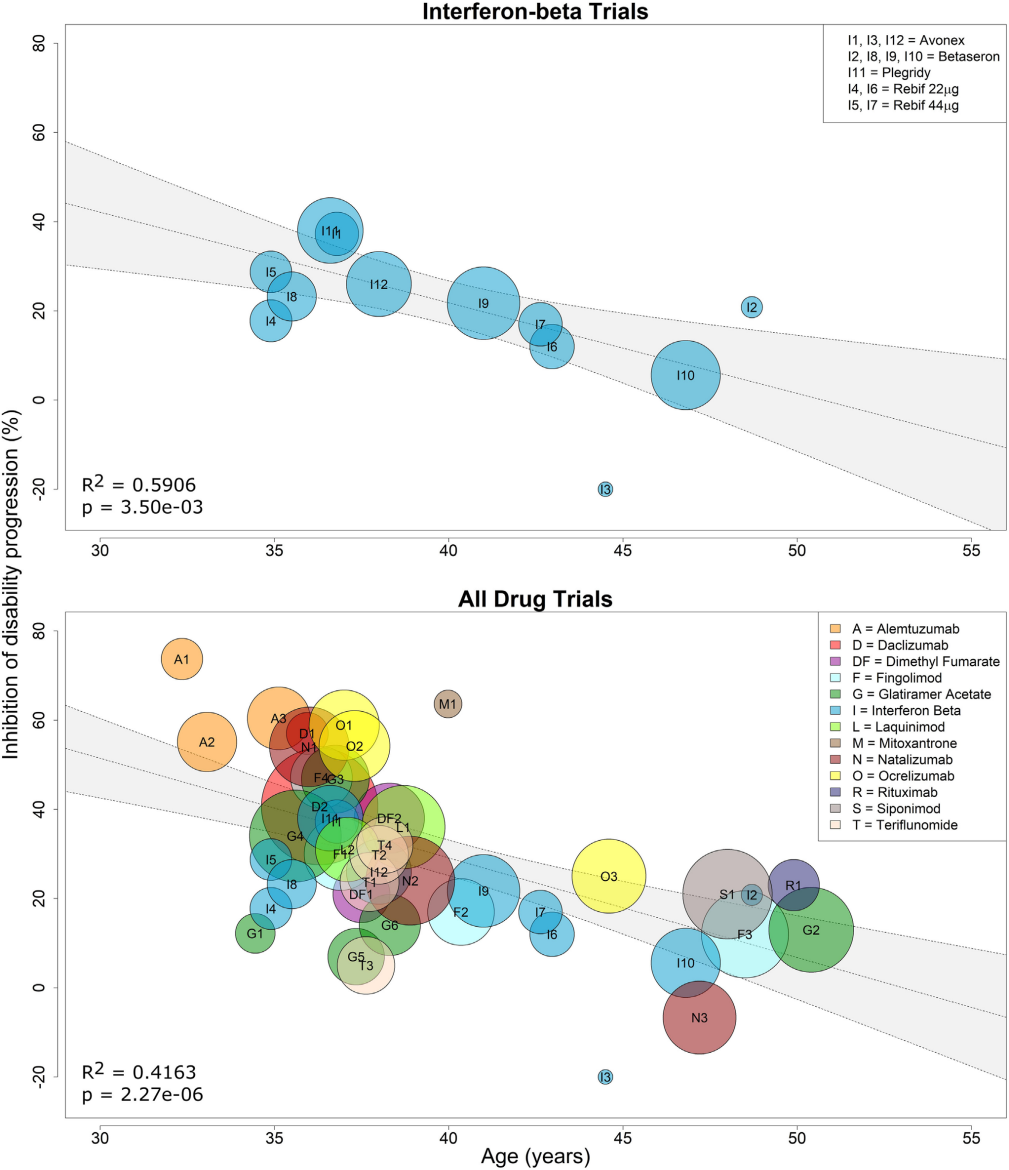
Efficacy of interferon-beta preparations and all immunomodulatory drugs on sustained disability progression decreases with age. Linear regression of the efficacy of all interferon-beta formulations against placebo on sustained disability progression as a function of age (top panel). Each contributing trial has assigned weight proportional to the number of subjects and trial duration (see Eq. 2 in Materials and Methods). The resulting linear regression was used to estimate percent inhibition of disability progression (%IDP) of interferon beta against placebo at baseline age (see Eq. 1). This estimate was then used to recalculate %IDP for all immunomodulatory drugs against placebo as a function of age (see Eq. 7). Linear regression of the efficacy of all drugs against placebo on sustained disability progression as a function of age (bottom panel). Again, each contributing trial has assigned weight proportional to the number of subjects and trial duration. The coefficient of determination (*R*^2^) and *p*-values are indicated in the respective plots, while the inset legends denote the trial indices.

Because interferon-beta preparations were used as active comparators in several clinical trials that lacked a placebo arm, we first performed a linear regression of the efficacy of all interferon-beta treatments against placebo as a function of age. We observed a negative relationship between %IDP and mean age, and the weighted regression model $\left({\hat{\text{IDP}}}_{W_{i}}=103.04-2.03{\text{Age}}_{i},\;\text{slope}\;95\%\;\mathrm{CI}=(-3.22,-0.84)\right)$ had strong evidence of a relationship, with age explaining approximately 59% of the variance in %IDP (*R*^2^ = 0.5906, two-tailed *p* = 0.0035; Figure 2, top panel). This model was used to impute efficacy for interferon-beta comparators in trials that lacked a placebo arm (see Materials and Methods).

**Figure 3:**
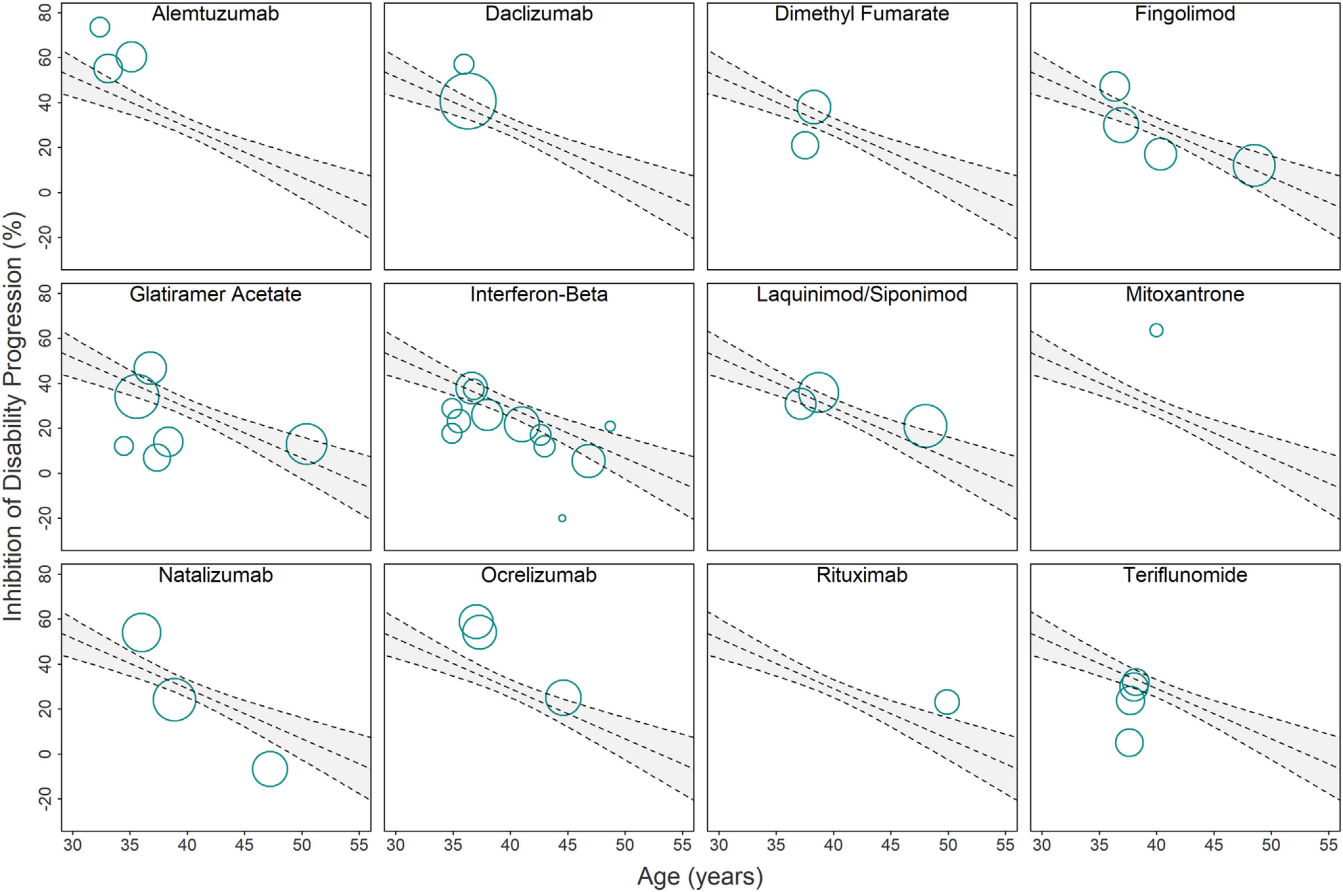
Relationship between immunomodulatory drugs and original linear regression model used for computing drug-specific weighted residuals. Due to the overlap of clinical trials in the Figure 2 (bottom panel) linear regression model, we provide a separate visual representation of all clinical trials that studied individual drugs or drug classes. Each circle corresponds to a single clinical trial with area proportional to the number of subjects and trial duration $\text{(weight}=\pi r^{2}\to r=\sqrt{\text{weight}/\pi }\text{,where }r\text{ is the radius of the circle)}$. The gray area depicts 95% confidence interval estimates. Trials with circle center above the regression line have better-than-average efficacy adjusted for age, while trials with circle center below the regression line have worse-than-average efficacy adjusted for age. The distances from the circle center to the regression line (i.e., residuals) are adjusted by weight and SD (see Eq. 8 in Materials and Methods) and then averaged to compute the drug-specific weighted residuals (Figures 4A,B).

Using these imputed data, we performed an analogous simple linear regression $\left({\hat{\text{IDP}}}_{W_{i}}=118.46-2.23{\text{Age}}_{i},\;\text{slope}\;95\%\;\mathrm{CI}=(-3.06,-1.41)\right)$ for all 38 clinical trials and found strong evidence of a linear relationship, with mean age explaining approximately 42% of the variance in %IDP (*R*^2^ = 0.4163, two-tailed *p* = 2.27e−06; Figure 2, bottom panel). However, we hypothesized that the model could be further improved by including a predictor that explained a patient’s average response to therapy. This response (therapeutic efficacy) was divided into categories of high and low based by examining the difference (residuals) between the observed and model-predicted efficacy for each DMT (Figure 3).

### IDP As Dependent on Age and Therapeutic Efficacy (Low versus High)

**Figure 4:**
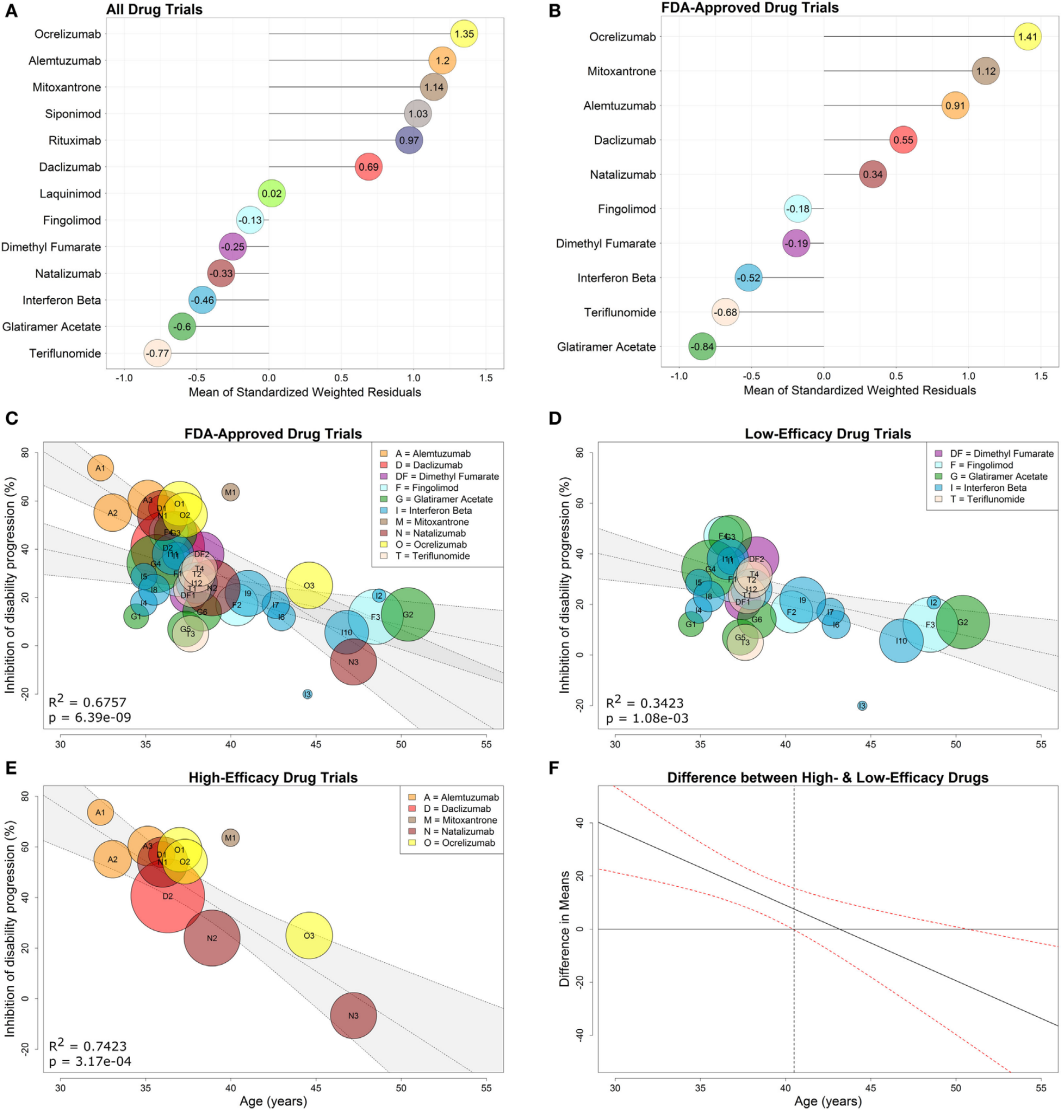
Low- and high-efficacy categories derived from drug-specific weighted residuals and development of optimized model with interaction between age and efficacy. Comparative efficacy ranks for standardized, drug-specific weighted residual means computed from the linear regression fit to all drugs **(A)** or fit to clinical trials of FDA-approved drugs studied in FDA-approved indications **(B)**. The means of the drug-specific residuals are provided directly in the lollipop plots. FDA-approved immunomodulatory disease-modifying therapies from **(B)** were then separated into high-efficacy drugs (i.e., drugs with positive means) and low-efficacy drugs (i.e., drugs with negative means). A regression model that includes all FDA-approved drugs with an interaction between age and efficacy (0 for low-efficacy, 1 for high-efficacy) is depicted in **(C)**. Simple weighted linear regressions were fit to clinical trials of low-efficacy **(D)** and high-efficacy **(E)** drugs using only trials that studied FDA-approved drugs. Corresponding coefficients of determination (*R*^2^) and *p*-values are included in the individual plots, while the inset legends provide color and alphabet code for individual drugs. **(F)** The 95% confidence interval denotes the statistically significant difference in means between low- and high-drug efficacy as a function of age. The gray dashed vertical line indicates that there is no significant difference between low- and high-efficacy drugs past age 40.5 years.

As described in Section “Materials and Methods,” we first calculated the weighted standardized residuals (Eq. 8) for each of the 13 drug types using the observed and model-predicted values (derived from a regression through all drug types). We then computed the mean of these residuals (Figure 4A) and used the sign (positive or negative) of each mean to dichotomize the drugs into high- or low-efficacy categories.

To model efficacies observed in clinical practice when prescribers administer FDA-approved drugs only for approved indications, we performed a separate analysis of drug efficacy in trials targeting only FDA-approved DMTs, studied in approved MS subtypes (i.e., progressive MS trials were included only if progressive MS is an FDA-approved indication, such as in the case of mitoxantrone and ocrelizumab). This included 10 drug types in 31 trials involving 20,466 patients. We again computed the mean of the weighted standardized residuals (Eq. 8) for each of the 10 drug types using the observed and model-predicted values (derived from a regression through the 10 drug types) (Figure 4B) and then dichotomized FDA-approved DMTs into low-efficacy (negative means) and high-efficacy (positive means). The low-efficacy drugs included fingolimod, dimethyl fumarate, all interferon-beta preparations, teriflunomide, and glatiramer acetate. The high-efficacy drugs included ocrelizumab, mitoxantrone, alemtuzumab, daclizumab, and natalizumab.

As discussed in Section “Materials and Methods,” to further explore whether baseline disability (measured by EDSS) had a measurable effect on DMT efficacy (dependent or independent of Age), we used a step-down procedure to examine potential interactions between Age, Efficacy (indicator coded 0 for low and 1 for high), and EDSS. We started with a model containing interactions between all three features (Age, EDSS, and Efficacy) and sequentially dropped the most complex terms that were determined to have the least significant (*p* > 0.01) leading coefficients. We reanalyzed the model at each step in this iterative process until we arrived at a model fit in which all coefficients were statistically significant. All reported *p*-values are rounded up when truncated.

Using a significance level of α = 0.01 and the step-down procedure outlined in Section “Materials and Methods,” the three-way interaction between Age, Efficacy, and EDSS was not found to be important $\left(t_{32}=-1.908,p=0.07\right)$. The interaction of Age and EDSS was then dropped $\left(t_{33}=-0.534,p=0.60\right)$, followed by the interaction of Efficacy and EDSS $\left(t_{34}=2.727,p=0.02\right)$, and then, finally, EDSS was dropped $\left(t_{35}=0.180,p=0.86\right)$, leaving a model with Age interacting with Efficacy.

The resulting interaction model:

(10) IDP^Wi=83.71−1.50(Agei)+122.69(Efficacyi)−2.84(Agei)(Efficacyi)

explained 68% of variance (*R*^2^ = 0.6757, overall *F*-test *p* = 6.39e−09; Figure 4C) with significant evidence of a difference in the relationship between age and disability inhibition based on efficacy categorization $\left(t_{36}=-3.46,p=0.002\right)$. This interaction model was a significant improvement over the initial regression of IDP versus Age where only 42% of the variance in IDP was explained by age [*R*^2^ = 0.6757 (interaction model) versus *R*^2^ = 0.4163 (simple model)].

After finding evidence for a difference in slopes for low- and high-efficacy drugs, we examined the two efficacy subgroups separately to obtain the following models:

(11) $$\text{Low}-\text{efficacy:}{\hat{\;\text{IDP}}}_{W_{i}}=83.71-1.50{\text{Age}}_{i},\text{ slope }95\%\;\mathrm{CI}=(-2.34,-0.66)$$

(12) High-efficacy:IDP^Wi=206.39−4.34Agei, slope 95% CI=(−6.15,−2.54)

We observed strong relationships between %IDP and Age, which explained 34% of variance in the low-efficacy subgroup (*R*^2^ = 0.3423, two-tailed *p* = 1.08e−03; Figure 4D) and 74% of variance in the high-efficacy subgroup (*R*^2^ = 0.7423, two-tailed *p* = 3.17e−04; Figure 4E).

It is evident from Figure 4C that the CIs for the regressions of low- and high-efficacy therapy eventually overlap. However, without appropriate statistical tests, it is not necessarily true that this overlap occurs at the correct location used to determine evidence of differences in the groups (28). Thus, we estimated the difference in means between low and high %IDP, and the resulting 95% CI (Figure 4F) suggests that this difference is present in younger patients up to age 40.5, after which point there is no apparent benefit to prescribing high-over low-efficacy therapy to an *average* patient.

## Discussion

This meta-analysis of randomized, blinded clinical trials of MS DMTs against placebo or active comparator demonstrated unequivocally that the efficacy of immunomodulatory DMTs decreases with age. Age predicts IDP by immunomodulatory DMTs more strongly than EDSS. In fact, in higher order models there were no significant three- or two-way interactions between EDSS and variables Age and/or Efficacy (indicator coded as 0 for low and 1 for high); instead, the optimized model consisted of interactions between only Age and Efficacy. The observation that Age and Efficacy jointly predict more than 67% of variance in disability progression highlights that age is a major modulator in the therapeutic efficacy of immunomodulatory drugs.

Before we discuss implications of our findings, we acknowledge the following limitations: (1) lack of trials with mean age <30 and >55 years; (2) all interferon-beta preparations were treated as equivalent to simplify the interferon beta versus placebo regression used for later imputation; (3) the assumption that drugs belonging to the same category (low- and high-efficacy) have comparable efficacies during the entire age-spectrum, which may or may not be correct; (4) trials that compared a new DMT to an active comparator generally disadvantaged the “older” drug. Because inclusion criteria required disease activity, these trials excluded patients who had done well on the comparator therapy (29); and (5) the comparative efficacy estimates for some drugs (e.g., mitoxantrone) are based on a single (and sometimes unusually small) trial and therefore may not be reliable.

These limitations are mostly based on lack of public access to raw datasets from clinical trials even after regulatory approval of tested drugs, and are thus beyond our control. Our study may add impetus to a debate as to whether regulatory agencies should demand publication of raw data from the trials that led to drug approval. Public access to such raw data would significantly strengthen meta-analyses and, in this particular study, would allow for better estimation of therapeutic efficacy for patients younger than 30 and older than 55 years. Whenever even partial age-based subgroup analyses from clinical trials of MS drugs were published (30–32), they were consistent with the results of this meta-analysis (i.e., younger patients always had higher efficacy than older patients, even though the difference may not have reached statistical significance because the trials were not powered for subgroup analyses). Other stated limitations are not linked to lack of raw data, but are still beyond our control. For example, while different mechanisms of action may make one drug (e.g., ocrelizumab) more efficacious in the later stages of MS than other drugs from the same category (e.g., natalizumab), a superiority hypothesis is currently untestable, because it requires prohibitively large cohorts. Thus, we also *caution* against over-interpretation of DMT efficacy rankings. While low- versus high-efficacy drug categories enhance the model, this meta-analysis does not provide sufficient power for superiority claims of one drug over another if they were not tested against each other directly in clinical trials. Nevertheless, the efficacy ranks given in Figure 4B are based entirely on clinical trial data adjusted for patient age, and thus, should be considered the most objective comparative efficacies currently available in the public domain.

Notwithstanding these limitations, our results inform the decision process when addressing common therapeutic dilemmas, such as the decision to initiate or delay high-potency treatments at an early age. Delaying any DMT, even for a few years, leads to a decrease in cumulative efficacy that cannot be easily regained by opting for more aggressive treatments at a later age. In the fourth decade of life, the efficacies of all DMTs overlap, and, after age 53, the model predicts no therapeutic benefit for the *average* patient. Interestingly, the upper age limit of 53 years extrapolated from meta-analysis regression models is close to the upper age limit of 55 years implemented in the inclusion criteria of the ocrelizumab (ORATORIO) PPMS trial (3), which was selected based on the age-based subgroup analyses of the rituximab (OLYMPUS) PPMS trial (33).

Thus, a prescribing clinician must consider the possibility that starting or continuing immunomodulatory DMT beyond age 53 will expose an *average* patient to treatment-associated risks with few, if any, potential benefits. The results of ASCEND trial (Table 1; Figure 3), in which more SPMS patients treated with natalizumab achieved sustained disability progression in comparison with placebo (although the difference did not reach statistical significance), should not be ignored. Rather, in view of this meta-analysis, it should serve as a reminder that aggressive immunomodulatory DMTs may be harmful in older MS patients, irrespective of cumulative side-effects. By limiting migration of immune cells to CNS tissue, drugs like natalizumab may block repair processes, including remyelination, facilitated by immune cells (34–36).

This meta-analysis does not suggest that all patients older than 53 should remain untreated. The model is based on *mean outcomes* within trial cohorts. Behind every *mean* lies a distribution (e.g., Gaussian), and where on that distribution a specific patient falls cannot be determined from group data as it likely depends on patient-specific genetic and environmental factors. Indeed, if a patient older than 53 has MS relapses and abundant contrast-enhancing lesions on CNS imaging, s/he is likely to receive higher than *average* benefit from immunomodulatory DMT. However, these types of patients are rare. If *every* patient older than 53 years is on immunomodulatory DMT, then this meta-analysis indicates that half of such patients are exposed to cumulative side-effects with little to no potential for therapeutic benefit. Similarly, we do not argue that every MS patient younger than 40.5 years should be started on high-efficacy therapy. Such a recommendation would ignore the fact that some patients have benign disease and may not accumulate substantial disability during a normal life-span. Unfortunately, the lack of validated models of MS severity that can identify patients with benign (or aggressive) MS with acceptable accuracy limits such personalized decisions. Without this central knowledge, the results of this meta-analysis suggest that patients younger than age 40.5, who choose to start low-efficacy DMTs, must be followed closely with clinical examinations and imaging and should be promptly switched to a high-efficacy DMT if/when they develop clinical or radiological evidence of disease activity.

A model of MS severity that can predict MS course with sufficient accuracy would spare patients with mild/benign MS the risks and side-effects of high-efficacy treatments. In fact, the attempt to develop an accurate model of MS severity (i.e., a model that can predict the future rates of accumulation of MS disability based on cross-sectional data) was the motivation for this study. We noticed that observational studies that investigate prognostic biomarkers, or aim to identify biological modifiers of MS severity, are seldom adjusted for the efficacy of administered treatments. Conversely, when treatments are used in complex statistical models as covariates, they do not seem to exert consistent effects. This is incompatible with clinical trial evidence that (some) DMTs exert reproducible efficacy on disability progression and prompted the hypothesis that efficacies of immunomodulatory DMTs are not stable, but instead, change with age. Therefore, observational studies that seek adjustments for efficacy of administered treatments must use a model that considers Efficacy and Age simultaneously.

Although we anticipate that the presented model will facilitate development of more accurate measures of MS severity than the currently available MS severity score (37), until such predictive models are validated, the astute clinician must merge information from group-based analyses with features gathered from a patient’s medical history, neurological examination, and auxiliary tests. We successfully used the graphs provided in this publication to inform discussions between clinicians and elderly MS patients regarding the appropriate timing of DMTs and to convince young patients to not wait until exhausting low-efficacy alternatives before initiating high-efficacy treatment. We expect that MS researchers, clinicians, and patients alike will find our results informative in this complex decision-making process.

## Author Contributions

BB was responsible for original study concept, model design, and study supervision. MT-M and AW were responsible for acquisition of data. AW, KJ, and MG were responsible for model design and statistical analyses. BB, AW, MT-M, and MG were responsible for drafting the text. All authors were responsible for revision and approval of the final manuscript.

## Conflict of Interest Statement

BB declares the following COI: she is co-inventor on several patents related to daclizumab therapy for MS and, as such, has received patent royalty payments from the NIH. The remaining authors have no competing interests to declare.

## Abbreviations

CDP, confirmed disability progression; CNS, central nervous system; CSF, cerebrospinal fluid; DMT, disease-modifying therapy; EDSS, expanded disability status scale; MS, multiple sclerosis; MSSS, multiple sclerosis severity score; %IDP, percent inhibition of disability progression; PPMS, primary-progressive multiple sclerosis; RRMS, relapsing-remitting multiple sclerosis; *R*^2^, coefficient of determination; SPMS, secondary-progressive multiple sclerosis.

## References

[1] Stys PK Zamponi GW van Minnen J Geurts JJ. Will the real multiple sclerosis please stand up? Nat Rev Neurosci (2012) 13:507–14. 10.1038/nrn3275 22714021

[2] Komori M Blake A Greenwood M Lin YC Kosa P Ghazali D CSF markers reveal intrathecal inflammation in progressive multiple sclerosis. Ann Neurol (2015) 78:3–20. 10.1002/ana.24408 25808056 PMC5568079

[3] Montalban X Hauser SL Kappos L Arnold D Bar-Or A Comi G Ocrelizumab versus placebo in primary progressive multiple sclerosis. N Engl J Med (2017) 376:209–20. 10.1056/NEJMoa1606468 28002688

[4] Lassmann H van Horssen J Mahad D. Progressive multiple sclerosis: pathology and pathogenesis. Nat Rev Neurol (2012) 8:647–56. 10.1038/nrneurol.2012.168 23007702

[5] Magliozzi R Howell O Vora A Serafini B Nicholas R Puopolo M Meningeal B-cell follicles in secondary progressive multiple sclerosis associate with early onset of disease and severe cortical pathology. Brain (2007) 130:1089–104. 10.1093/brain/awm038 17438020

[6] Magliozzi R Howell OW Reeves C Roncaroli F Nicholas R Serafini B A gradient of neuronal loss and meningeal inflammation in multiple sclerosis. Ann Neurol (2010) 68:477–93. 10.1002/ana.22230 20976767

[7] Choi SR Howell OW Carassiti D Magliozzi R Gveric D Muraro PA Meningeal inflammation plays a role in the pathology of primary progressive multiple sclerosis. Brain (2012) 135:2925–37. 10.1093/brain/aws189 22907116

[8] Komori M Lin YC Cortese I Blake A Ohayon J Cherup J Insufficient disease inhibition by intrathecal rituximab in progressive multiple sclerosis. Ann Clin Transl Neurol (2016) 3:166–79. 10.1002/acn3.293 27042677 PMC4774261

[9] Komori M Kosa P Stein J Zhao V Blake A Cherup J Pharmacodynamic effects of daclizumab in the intrathecal compartment. Ann Clin Transl Neurol (2017) 4:478–90. 10.1002/acn3.427 28695148 PMC5497534

[10] Wuest SC Mexhitaj I Chai NR Romm E Scheffel J Xu B A complex role of herpes viruses in the disease process of multiple sclerosis. PLoS One (2014) 9:e105434. 10.1371/journal.pone.0105434 25148387 PMC4141762

[11] Campbell GR Ziabreva I Reeve AK Krishnan KJ Reynolds R Howell O Mitochondrial DNA deletions and neurodegeneration in multiple sclerosis. Ann Neurol (2011) 69:481–92. 10.1002/ana.22109 21446022 PMC3580047

[12] Fischer MT Sharma R Lim JL Haider L Frischer JM Drexhage J NADPH oxidase expression in active multiple sclerosis lesions in relation to oxidative tissue damage and mitochondrial injury. Brain (2012) 135:886–99. 10.1093/brain/aws012 22366799 PMC3286337

[13] Trapp BD Stys PK. Virtual hypoxia and chronic necrosis of demyelinated axons in multiple sclerosis. Lancet Neurol (2009) 8:280–91. 10.1016/S1474-4422(09)70043-2 19233038

[14] Yang R Dunn JF. Reduced cortical microvascular oxygenation in multiple sclerosis: a blinded, case-controlled study using a novel quantitative near-infrared spectroscopy method. Sci Rep (2015) 5:16477. 10.1038/srep16477 26563581 PMC4643232

[15] Lassmann H Reindl M Rauschka H Berger J Aboul-Enein F Berger T A new paraclinical CSF marker for hypoxia-like tissue damage in multiple sclerosis lesions. Brain (2003) 126:1347–57. 10.1093/brain/awg127 12764056

[16] McMahon JM McQuaid S Reynolds R FitzGerald UF. Increased expression of ER stress- and hypoxia-associated molecules in grey matter lesions in multiple sclerosis. Mult Scler (2012) 18:1437–47. 10.1177/1352458512438455 22354737

[17] Liddelow SA Guttenplan KA Clarke LE Bennett FC Bohlen CJ Schirmer L Neurotoxic reactive astrocytes are induced by activated microglia. Nature (2017) 541:481–7. 10.1038/nature21029 28099414 PMC5404890

[18] International Multiple Sclerosis Genetics Consortium, Wellcome Trust Case Control Consortium 2 Sawcer S Hellenthal G Pirinen M Spencer CC Genetic risk and a primary role for cell-mediated immune mechanisms in multiple sclerosis. Nature (2011) 476:214–9. 10.1038/nature10251 21833088 PMC3182531

[19] Confavreux C Vukusic S. Age at disability milestones in multiple sclerosis. Brain (2006) 129:595–605. 10.1093/brain/awl313 16415309

[20] Confavreux C Vukusic S. Natural history of multiple sclerosis: a unifying concept. Brain (2006) 129:606–16. 10.1093/brain/awl313 16415308

[21] Moher D Liberati A Tetzlaff J Altman DG Group P Preferred reporting items for systematic reviews and meta-analyses: the PRISMA statement. PLoS Med (2009) 6:e1000097 10.1371/journal.pmed.1000097 19621072 PMC2707599

[22] Sormani MP Bonzano L Roccatagliata L Cutter GR Mancardi GL Bruzzi P. Magnetic resonance imaging as a potential surrogate for relapses in multiple sclerosis: a meta-analytic approach. Ann Neurol (2009) 65:268–75. 10.1002/ana.21606 19334061

[23] Sormani MP Arnold DL De Stefano N. Treatment effect on brain atrophy correlates with treatment effect on disability in multiple sclerosis. Ann Neurol (2014) 75:43–9. 10.1002/ana.24018 24006277

[24] Fox RJ Miller DH Phillips JT Hutchinson M Havrdova E Kita M Placebo-controlled phase 3 study of oral BG-12 or glatiramer in multiple sclerosis. N Engl J Med (2012) 367:1087–97. 10.1056/NEJMoa1206328 22992072

[25] R: A language and environment for statistical computing [computer program]. Vienna, Austria: R Foundation for Statistical Computing (2016).

[26] RStudio: Integrated Development for R. [computer program]. Boston, MA: RStudio, Inc., (2015).

[27] Sheather SA Modern Approach to Regression with R. New York: Springer (2009).

[28] Knol MJ Pestman WR Grobbee DE The (mis)use of overlap of confidence intervals to assess effect modification. Eur J Epidemiol (2011) 26:253–4. 10.1007/s10654-011-9563-8 21424218 PMC3088813

[29] Bielekova B Tintore M Sustained reduction of MS disability: new player in comparing disease-modifying treatments. Neurology (2016) 87:1966–7. 10.1212/WNL.0000000000003314 27733564

[30] Miller AE O’Connor P Wolinsky JS Confavreux C Kappos L Olsson TP Pre-specified subgroup analyses of a placebo-controlled phase III trial (TEMSO) of oral teriflunomide in relapsing multiple sclerosis. Mult Scler (2012) 18:1625–32. 10.1177/1352458512450354 22723573 PMC3573676

[31] Hutchinson M Kappos L Calabresi PA Confavreux C Giovannoni G Galetta SL The efficacy of natalizumab in patients with relapsing multiple sclerosis: subgroup analyses of AFFIRM and SENTINEL. J Neurol (2009) 256:405–15. 10.1007/s00415-009-0093-1 19308305

[32] Devonshire V Havrdova E Radue EW O’Connor P Zhang-Auberson L Agoropoulou C Relapse and disability outcomes in patients with multiple sclerosis treated with fingolimod: subgroup analyses of the double-blind, randomised, placebo-controlled FREEDOMS study. Lancet Neurol (2012) 11:420–8. 10.1016/S1474-4422(12)70056-X 22494956

[33] Hawker K O’Connor P Freedman MS Calabresi PA Antel J Simon J Rituximab in patients with primary progressive multiple sclerosis: results of a randomized double-blind placebo-controlled multicenter trial. Ann Neurol (2009) 66:460–71. 10.1002/ana.21867 19847908

[34] Schwartz M “Tissue-repairing” blood-derived macrophages are essential for healing of the injured spinal cord: from skin-activated macrophages to infiltrating blood-derived cells? Brain Behav Immun (2010) 24:1054–7. 10.1016/j.bbi.2010.01.010 20149864

[35] Kipnis J Cohen H Cardon M Ziv Y Schwartz M. T cell deficiency leads to cognitive dysfunction: implications for therapeutic vaccination for schizophrenia and other psychiatric conditions. Proc Natl Acad Sci U S A (2004) 101:8180–5. 10.1073/pnas.0402268101 15141078 PMC419577

[36] Bieber AJ Kerr S Rodriguez M. Efficient central nervous system remyelination requires T cells. Ann Neurol (2003) 53:680–4. 10.1002/ana.10578 12731006

[37] Roxburgh RH Seaman SR Masterman T Hensiek AE Sawcer SJ Vukusic S Multiple Sclerosis Severity Score: using disability and disease duration to rate disease severity. Neurology (2005) 64:1144–51. 10.1212/01.WNL.0000156155.19270.F8 15824338

