# Supplementary material for: Meta-analysis of the Age-Dependent Efficacy of Multiple Sclerosis Treatments
Source: Front Neurol. 2017 Nov 10;8:577. doi: 10.3389/fneur.2017.00577 (PMC5686062; doi:10.3389/fneur.2017.00577)
Supplement: Supplementary file 1 [file data_sheet_1.pdf]

Supplementary material

TEXT FILES

Instructions to user: Paste the following text files into Notepad (or other text editor) and save according to the indicated names. These files are necessary to run the following R code and must be saved in the same directory as your code.

Save as data_table_1.txt

Study	DI	Age	Weight	Dose	N	Drug
I1	37.24928	36.79502	425.67828	1.00000	301.00000	I1
I2	20.83333	48.69863	103.23759	1.00000	73.00000	I2
I3	-20.00000	44.50000	49.49747	1.00000	35.00000	I3
I4	17.76266	34.90000	399.51533	1.00000	282.50000	I4
I5	28.72671	34.90000	392.44426	1.00000	277.50000	I5
I6	12.00000	42.96838	447.73513	1.00000	258.50000	I6
I7	17.00000	42.63344	428.68257	1.00000	247.50000	I7
I8	23.21429	35.50000	545.60059	1.00000	244.00000	I8
I9	21.73038	41.00028	1190.66830	1.00000	718.00000	I9
I10	5.58895	46.80000	1082.53175	1.00000	625.00000	I10
I11	38.00000	36.60356	970.96492	1.00000	1012.00000	I11
I12	26.00000	38.00000	950.35151	1.00000	672.00000	I12

Save as data_table_2.txt

Study	DI	Age	Weight	Type	N	Drug	Factors
A1	73.6852475219	32.3479820628	386.2473300879	r	223.00	A1	A
A2	55.1208108605	33.0664298401	796.2022356161	r	563.00	A2	A
A3	60.3937236110	35.1216560510	888.1261171703	r	628.00	A3	A
D1	57.0000000000	35.9436893204	397.0000000000	r	397.00	D1	D
D2	40.6294549591	36.2998370451	3059.4083231384	r	1841.00	D2	D
DF1	21.0000000000	37.4977798335	733.3878725639	r	540.50	DF1	DF
DF2	38.0000000000	38.2995110024	1108.5622421548	r	817.00	DF2	DF
F1	30.0000000000	36.8975088968	1192.1820330805	r	843.00	F1	F
F2	17.0000000000	40.3510518934	1001.0612848865	r	713.00	F2	F
F3	11.9900000000	48.5000000000	1709.5960415147	p	823.00	F3	F

F4	47.1406277608	36.3483833718	860.0000000000	r	860.00	F4	F
G1	12.1951219512	34.4494023904	354.9676041556	r	251.00	G1	G
G2	13.0000000000	50.4000000000	1619.4675050769	p	935.00	G2	G
G3	46.7646964277	36.7494764398	1036.6481676943	r	764.00	G3	G
G4	34.0396567967	35.6001486989	1899.2888142671	r	1343.00	G4	G
G5	7.0000000000	37.3417952314	721.1760485989	r	531.50	G5	G
G6	14.0762708917	38.3123772102	843.5087432860	r	487.00	G6	G
I1	37.2492836676	36.7950166113	425.6782822743	r	301.00	I1	I
I2	20.8333333333	48.6986301370	103.2375900532	p	73.00	I2	I
I3	-20.0000000000	44.5000000000	49.4974746831	p	35.00	I3	I
I4	17.7626606198	34.9000000000	399.5153313704	r	282.50	I4	I
I5	28.7267080745	34.9000000000	392.4442635585	r	277.50	I5	I
I6	12.0000000000	42.9683788122	447.7351337566	p	258.50	I6	I
I7	17.0000000000	42.6334420881	428.6825748733	p	247.50	I7	I
I8	23.2142857143	35.5000000000	545.6005865099	r	244.00	I8	I
I9	21.7303822938	41.0002785515	1190.6682997376	p	718.00	I9	I
I10	5.5889530385	46.8000000000	1082.5317547306	p	625.00	I10	I
I11	38.0000000000	36.6035573123	970.9649220815	r	1012.00	I11	I
I12	26.0000000000	38.0000000000	950.3515139147	r	672.00	I12	I
L1	36.0000000000	38.7000000000	1564.1201999846	r	1106.00	L1	L
L2	31.0000000000	37.1000000000	931.9667376039	r	659.00	L2	L
M1	63.6400000000	39.9812903226	175.3624817343	rp	124.00	M1	M
N1	54.0000000000	36.0000000000	1429.0385189501	r	942.00	N1	N
N2	24.0000000000	38.9000000000	1776.4374795016	r	1171.00	N2	N
N3	-6.6666666667	47.2000000000	1203.5430952158	p	887.00	N3	N
O1	58.9025459068	36.9998781973	1113.9897194726	r	821.00	O1	O
O2	54.1923860092	37.3001197605	1132.9858900848	r	835.00	O2	O
O3	25.0000000000	44.6000000000	1234.0700344794	p	731.00	O3	O
R1	23.0000000000	49.9000000000	595.6656356254	p	439.00	R1	R
S1	21.0000000000	48.0000000000	1803.0795184905	p	1363.00	S1	S
T1	23.7000000000	37.7315068493	786.5103704311	r	546.50	T1	T
T2	29.8000000000	38.0014801110	776.4361296388	r	539.50	T2	T
T3	5.0000000000	37.6259751037	746.8718115113	r	601.00	T3	T
T4	32.0000000000	38.1656663725	714.1398730832	r	564.00	T4	T

Save as data_table_3.txt

Study	DI	Age	Weight	Type	N	Drug	Efficacy	Factors
A1	73.68524752	32.34798206	386.2473301	r	223	A1	2	A
A2	55.12081086	33.06642984	796.2022356	r	563	A2	2	A
A3	60.39372361	35.12165605	888.1261172	r	628	A3	2	A
D1	57	35.94368932	397	r	397	D1	2	D
D2	40.62945496	36.29983705	3059.408323	r	1841	D2	2	D
DF1	21	37.49777983	733.3878726	r	540.5	DF1	1	DF
DF2	38	38.299511	1108.562242	r	817	DF2	1	DF
F1	30	36.8975089	1192.182033	r	843	F1	1	F
F2	17	40.35105189	1001.061285	r	713	F2	1	F
F3	11.99	48.5	1709.596042	p	823	F3	1	F
F4	47.14062776	36.34838337	860	r	860	F4	1	F
G1	12.19512195	34.44940239	354.9676042	r	251	G1	1	G
G2	13	50.4	1619.467505	p	935	G2	1	G
G3	46.76469643	36.74947644	1036.648168	r	764	G3	1	G
G4	34.0396568	35.6001487	1899.288814	r	1343	G4	1	G
G5	7	37.34179523	721.1760486	r	531.5	G5	1	G
G6	14.07627089	38.31237721	843.5087433	r	487	G6	1	G
I1	37.24928367	36.79501661	425.6782823	r	301	I1	1	I
I2	20.83333333	48.69863014	103.2375901	p	73	I2	1	I
I3	-20	44.5	49.49747468	p	35	I3	1	I
I4	17.76266062	34.9	399.5153314	r	282.5	I4	1	I
I5	28.72670807	34.9	392.4442636	r	277.5	I5	1	I
I6	12	42.96837881	447.7351338	p	258.5	I6	1	I
I7	17	42.63344209	428.6825749	p	247.5	I7	1	I
I8	23.21428571	35.5	545.6005865	r	244	I8	1	I
I9	21.73038229	41.00027855	1190.6683	p	718	I9	1	I
I10	5.588953039	46.8	1082.531755	p	625	I10	1	I
I11	38	36.60355731	970.9649221	r	1012	I11	1	I
I12	26	38	950.3515139	r	672	I12	1	I
M1	63.64	39.98129032	175.3624817	rp	124	M1	2	M
N1	54	36	1429.038519	r	942	N1	2	N
N2	24	38.9	1776.43748	r	1171	N2	2	N
N3	-6.666666667	47.2	1203.543095	p	887	N3	2	N
O1	58.90254591	36.9998782	1113.989719	r	821	O1	2	O
O2	54.19238601	37.30011976	1132.98589	r	835	O2	2	O
O3	25	44.6	1234.070034	p	731	O3	2	O

T1	23.7	37.73150685	786.5103704	r	546.5	T1	1		T
T2	29.8	38.00148011	776.4361296	r	539.5	T2	1		T
T3	5	37.6259751	746.8718115	r	601	T3	1		T
T4	32	38.16566637	714.1398731	r	564	T4	1		T

Save as data_table_miss.txt

Study	DI	Age	EDSS	Weight	Type	N	Drug	Efficacy	Factors
A1	73.68524752	32.34798206	1.9	386.2473301	r	223	A1	2	A
A2	55.12081086	33.06642984	2	796.2022356	r	563	A2	2	A
A3	60.39372361	35.12165605	2.7	888.1261172	r	628	A3	2	A
D1	57	35.94368932	2.750485437	397	r	397	D1	2	D
D2	40.62945496	36.29983705	2.5	3059.408323	r	1841	D2	2	D
DF1	21	37.49777983	2.6	733.3878726	r	540.5	DF1	1	DF
DF2	38	38.299511	2.4399022	1108.562242	r	817	DF2	1	DF
F1	30	36.8975089	2.399169632	1192.182033	r	843	F1	1	F
F2	17	40.35105189	2.4	1001.061285	r	713	F2	1	F
F3	11.99	48.5	4.67	1709.596042	p	823	F3	1	F
F4	47.14062776	36.34838337	2.214884527	860	r	860	F4	1	F
G1	12.19512195	34.44940239	2.599203187	354.9676042	r	251	G1	1	G
G2	13	50.4	4.9	1619.467505	p	935	G2	1	G
G3	46.76469643	36.74947644	2.340104712	1036.648168	r	764	G3	1	G
G4	34.0396568	35.6001487	2.326684015	1899.288814	r	1343	G4	1	G
G5	7	37.34179523	2.6	721.1760486	r	531.5	G5	1	G
G6	14.07627089	38.31237721	1.949115914	843.5087433	r	487	G6	1	G
I1	37.24928367	36.79501661	2.352491694	425.6782823	r	301	I1	1	I
I2	20.83333333	48.69863014	5.249315068	103.2375901	p	73	I2	1	I
I3	-20	44.5	5.25	49.49747468	p	35	I3	1	I
I4	17.76266062	34.9	2.466902655	399.5153314	r	282.5	I4	1	I
I5	28.72670807	34.9	2.466306306	392.4442636	r	277.5	I5	1	I
I6	12	42.96837881	5.467094703	447.7351338	p	258.5	I6	1	I
I7	17	42.63344209	5.333442088	428.6825749	p	247.5	I7	1	I
I8	23.21428571	35.5	2.9	545.6005865	r	244	I8	1	I
I9	21.73038229	41.00027855	5.149860724	1190.6683	p	718	I9	1	I
I10	5.588953039	46.8	5.15072	1082.531755	p	625	I10	1	I
I11	38	36.60355731	2.455177866	970.9649221	r	1012	I11	1	I
I12	26	38	2.5	950.3515139	r	672	I12	1	I
L1	36	38.7	2.6	1564.1202	r	1106	L1	NA	L
L2	31	37.1	2.5	931.9667376	r	659	L2	NA	L

M1	63.64	39.98129032	4.573870968	175.3624817	rp	124	M1	2		M
N1	54	36	2.3	1429.038519	r	942	N1	2		N
N2	24	38.9	2.4	1776.43748	r	1171	N2	2		N
N3	-6.666666667	47.2	5.2	1203.543095	p	887	N3	2		N
O1	58.90254591	36.9998782	2.804933009	1113.989719	r	821	O1	2		O
O2	54.19238601	37.30011976	2.810035928	1132.98589	r	835	O2	2		O
O3	25	44.6	4.7	1234.070034	p	731	O3	2		O
R1	23	49.9	4.8	595.6656356	p	439	R1	NA		R
S1	21	48	5.4	1803.079518	p	1363	S1	NA		S
T1	23.7	37.73150685	2.68	786.5103704	r	546.5	T1	1		T
T2	29.8	38.00148011	2.673358002	776.4361296	r	539.5	T2	1		T
T3	5	37.6259751	2.703543568	746.8718115	r	601	T3	1		T
T4	32	38.16566637	2.703133274	714.1398731	r	564	T4	1		T

Save as data_table_FDA_approve.txt

Study	DI	Age	Weight	Type	N	Drug	Efficacy	Factors
A1	73.68524752	32.34798206	386.2473301	r	223	A1	2	A
A2	55.12081086	33.06642984	796.2022356	r	563	A2	2	A
A3	60.39372361	35.12165605	888.1261172	r	628	A3	2	A
D1	57	35.94368932	397	r	397	D1	2	D
D2	40.62945496	36.29983705	3059.408323	r	1841	D2	2	D
DF1	21	37.49777983	733.3878726	r	540.5	DF1	1	DF
DF2	38	38.299511	1108.562242	r	817	DF2	1	DF
F1	30	36.8975089	1192.182033	r	843	F1	1	F
F2	17	40.35105189	1001.061285	r	713	F2	1	F
F4	47.14062776	36.34838337	860	r	860	F4	1	F
G1	12.19512195	34.44940239	354.9676042	r	251	G1	1	G
G3	46.76469643	36.74947644	1036.648168	r	764	G3	1	G
G4	34.0396568	35.6001487	1899.288814	r	1343	G4	1	G
G5	7	37.34179523	721.1760486	r	531.5	G5	1	G
G6	14.07627089	38.31237721	843.5087433	r	487	G6	1	G
I1	37.24928367	36.79501661	425.6782823	r	301	I1	1	I
I4	17.76266062	34.9	399.5153314	r	282.5	I4	1	I
I5	28.72670807	34.9	392.4442636	r	277.5	I5	1	I
I8	23.21428571	35.5	545.6005865	r	244	I8	1	I
I11	38	36.60355731	970.9649221	r	1012	I11	1	I
I12	26	38	950.3515139	r	672	I12	1	I
M1	63.64	39.98129032	175.3624817	rp	124	M1	2	M

N1	54	36	1429.038519	r	942	N1	2	N
N2	24	38.9	1776.43748	r	1171	N2	2	N
O1	58.90254591	36.9998782	1113.989719	r	821	O1	2	O
O2	54.19238601	37.30011976	1132.98589	r	835	O2	2	O
O3	25	44.6	1234.070034	p	731	O3	2	O
T1	23.7	37.73150685	786.5103704	r	546.5	T1	1	T
T2	29.8	38.00148011	776.4361296	r	539.5	T2	1	T
T3	5	37.6259751	746.8718115	r	601	T3	1	T
T4	32	38.16566637	714.1398731	r	564	T4	1	T

R CODE

(run in conjunction with above text files)

```
#-----------------------------------------------------------------------------------------------------
# Weighted Regression Analysis for Interferon-Beta, Low Efficacy, and High Efficacy Therapies
# Written by: Ann Marie Weideman (NINDS), Kory R Johnson, Ph.D. (NINDS), Mark Greenwood (MSU)
# Date:  20170512
# Revised: 20170929
# Email: annmarie.weideman@nih.gov, johnsonko@ninds.nih.gov, greenwood.stat@gmail.com
#-----------------------------------------------------------------------------------------------------

#install necessary packages (comment out after first run)
#install.packages("stats")
#install.packages("lattice")
#install.packages("effects")
#install.packages("interplot")
#install.packages("grid")

rm(list=ls())
options(object.size=Inf)

library(stats, quietly=T)
library(lattice, quietly=T)
library(effects, quietly=T)
library(interplot,quietly=T)
library(grid, quietly=T)
library(RColorBrewer)
library(ggplot2)

#import data table 1 (interferon-beta trials)
ddd1 <- read.table("data_table_1.txt",header=TRUE,row.names=1)

#import data table 2 (all trials)
ddd2 <- read.table("data_table_2.txt",header=TRUE,row.names=1)
```

```r
#import data table miss (trials with missing efficacy data coded as NA)
ddd.miss<-read.table("data_table_miss.txt",header=TRUE,row.names=1)

#import data table FDA approval (only FDA approved medications for approved MS subtypes)
ddd.approve<-read.table("data_table_FDA_approve.txt",header=TRUE,row.names=1)

#import data table 3 (trials for low/high efficacy plots - excludes Liquinimod, Rituximab, Siponimod)
ddd3 <-read.table("data_table_3.txt",header=TRUE,row.names=1)
ddd3.high <- ddd3[ddd3$Efficacy==2,] #high efficacy trials
ddd3.low <- ddd3[ddd3$Efficacy==1,] #low efficacy trials

#-----------------------------------------------------------------------------------------------------------
#Define color palettes
#-----------------------------------------------------------------------------------------------------------

#bubble colors for idebenone trials with transparency (alpha=0.5)
color.idebenone<-adjustcolor(rep("deepskyblue3",12),alpha=0.5)

#bubble colors for all trials with transparency (alpha=0.5)
color.all<-
adjustcolor(c(rep("darkorange",3),rep("red",2),rep("darkmagenta",2),rep("darkslategray1",4),rep("green4",6),
             rep("deepskyblue3",12),rep("lawngreen",2),"tan4",rep("darkred",3),rep("yellow1",3),
             "midnightblue","mistyrose4",rep("peachpuff",4)),alpha=0.5)

#bubble colors for FDA-approved therapies with transparency (alpha=0.5)
color.approve<-
adjustcolor(c(rep("darkorange",3),rep("red",2),rep("darkmagenta",2),rep("darkslategray1",4),rep("green4",6),
                             rep("deepskyblue3",12),"tan4",rep("darkred",3),rep("yellow1",3),
                             rep("peachpuff",4)),alpha=0.5)

#bubble colors for low efficacy therapies with transparency (alpha=0.5)
color.low<-adjustcolor(c(rep("darkmagenta",2),rep("darkslategray1",4),rep("green4",6),
                         rep("deepskyblue3",12),rep("peachpuff",4)),alpha=0.5)

#bubble colors for high efficacy therapies with transparency (alpha=0.5)
color.high<-adjustcolor(c(rep("darkorange",3),rep("red",2),"tan4",
                          rep("darkred",3),rep("yellow1",3)),alpha=0.5)

#legend color scheme for all therapies with transparency (alpha=0.5)
legend.all<-adjustcolor(c("darkorange","red","darkmagenta","darkslategray1","green4",
                            "deepskyblue3","lawngreen","tan4","darkred","yellow1",
                            "midnightblue","mistyrose4","peachpuff"),alpha=0.5)

#legend color scheme for FDA-approved therapies with transparency (alpha=0.5)
legend.approve<-adjustcolor(c("darkorange","red","darkmagenta","darkslategray1","green4",
                          "deepskyblue3","tan4","darkred","yellow1","peachpuff"),alpha=0.5)
```

```r
#legend color scheme for high efficacy therapies with transparency (alpha=0.5)
legend.high<-adjustcolor(c("darkorange","red","tan4","darkred","yellow1"),alpha=0.5)

#legend color scheme for low efficacy therapies with transparency (alpha=0.5)
legend.low<-adjustcolor(c("darkmagenta","darkslategray1","green4", "deepskyblue3","peachpuff"),alpha=0.5)


#-----------------------------------------------------------------------------------------------------
#Bubble plot for all interferon-beta trials (Figure 2A)
#-----------------------------------------------------------------------------------------------------

#set for export
jpeg("Plot_IFN_all_fit.jpg", width = 27, height = 31, units = 'in', res = 300)
par(mfrow = c(2, 1),
    oma = c(3, 4, 1, 1),
    mar = c(7, 7, 4, 2),
    mgp = c(5, 2.5, 1))

#create graphing parameters
xrange <- range(c(30,55))
yrange <- range(c(-25,80))
newx.df <- seq(-100,100, length.out=1000)
temp <- data.frame(x=newx.df)
dimnames(temp)[[2]][1] <- "Age"

#fit linear model to data (%Inhibition of Disability Progression (DI) against Age)
ddd1.fit <- lm(DI~Age,data=ddd1,weights=Weight)

#predict confidence intervals
preds.ddd1 <- predict(ddd1.fit,newdata=temp,interval='confidence')

#create plot window for data
plot(xrange, yrange,type = 'n',ylab="",
     xlab="",main="Interferon-beta Trials",cex.axis=2.5,cex.lab=3.25,cex.main=3.5)

#legend for various Interferon Betas inset in graph
legend("topright",
       c("I1, I3, I12 = Avonex","I2, I8, I9, I10 = Betaseron","I11 = Plegridy",
       expression(paste("I4, I6 = Rebif 22", mu,"g")),
       expression(paste("I5, I7 = Rebif 44", mu,"g"))), cex=2.1)

#shared y-axis title
mtext("Inhibition of disability progression (%)",side=2,line=-1,outer=T,cex=3.25)

#draw shaded region of confidence band
polygon(c(rev(newx.df), newx.df), c(rev(preds.ddd1[ ,3]), preds.ddd1[ ,2]), col=rgb(0, 0, 0,0.05), border = NA)
```

```r
#add best fit line
abline(coef(ddd1.fit),lty=2)

#draw dashed lines for confidence bands
lines(newx.df, preds.ddd1[ ,3], lty = 'dashed', col = 'black')
lines(newx.df, preds.ddd1[ ,2], lty = 'dashed', col = 'black')

par(new=T)

#draw bubbles using the fact that area = pi*r^2
with(ddd1, symbols(x=Age, y=DI, circles=sqrt(Weight/pi),
                   inches=1.5*sqrt(max(Weight)/max(ddd2$Weight)), ann=F,bg=color.idebenone,
                   xlim = xrange, ylim=yrange, xlab = "", ylab = "", xaxt='n', yaxt='n', pch=16,lwd=1.5))

par(new=T)

#ID labels
text(ddd1$Age,ddd1$DI,dimnames(ddd1)[[1]], cex=2)

box()

#------------------------------------------------------------
#Bubble plot for simple model (without interaction term)
#used to divide drugs into low and high efficacy categories
#(Figure 2B)
#------------------------------------------------------------

#fit weighted linear regression (%Inhibition of Disability Progression (DI) against Age)
ddd2.both.fit <- lm(DI~Age,data=ddd2,weights=Weight) #all drugs
ddd.approve.fit <- lm(DI~Age,data=ddd.approve,weights=Weight) #only FDA-approved drugs (subset of all drugs)

#predict confidence intervals
preds.ddd2 <- predict(ddd2.both.fit,newdata=temp,interval='confidence')

#create plot window for data
plot(xrange, yrange,type = 'n',ylab="",
     xlab="Age (years)",main="All Drug Trials",cex.axis=2.5,cex.lab=3.25,cex.main=3.5)

#legend for all drugs inset in graph
legend("topright",
       c("A = Alemtuzumab","D = Daclizumab","DF = Dimethyl Fumarate","F = Fingolimod","G = Glatiramer Acetate",
    "I = Interferon Beta", "L = Laquinimod", "M = Mitoxantrone", "N = Natalizumab","O = Ocrelizumab", "R = Rituximab",
         "S = Siponimod", "T = Teriflunomide"), fill=legend.all, cex=2.1)

#draw shaded region of confidence band
polygon(c(rev(newx.df), newx.df), c(rev(preds.ddd2[ ,3]), preds.ddd2[ ,2]),
```

```
                                        col=rgb(0, 0, 0,0.05), border = NA)

#add best fit line
abline(coef(ddd2.both.fit),lty=2)

#draw dashed lines for confidence bands
lines(newx.df, preds.ddd2[ ,3], lty = 'dashed', col = 'black')
lines(newx.df, preds.ddd2[ ,2], lty = 'dashed', col = 'black')

par(new=T)

#draw bubbles using the fact that area = pi*r^2
with(ddd2, symbols(x=Age, y=DI, circles=sqrt(Weight/pi),
                   inches=1.5, ann=F, bg=color.all,
                   xlim = xrange, ylim=yrange, xlab = "", ylab = "", xaxt='n', yaxt='n',
                   pch=16,lwd=1.5))

par(new=T)

#ID labels
text(ddd2$Age,ddd2$DI,dimnames(ddd2)[[1]], cex=2)

dev.off()

#-------------------------------------------------------------------
#Grid for individual regressions (Figure 3)
#-------------------------------------------------------------------

tiff("Plot_Grid.tiff", width = 12, height = 8, units = 'in', res = 300,compression = "lzw")

type.f = factor(ddd2$Factors,labels=c("Alemtuzumab", "Daclizumab","Dimethyl Fumarate","Fingolimod",
                                       "Glatiramer Acetate","Interferon-Beta","Laquinimod","Mitoxantrone",
                                       "Natalizumab","Ocrelizumab","Rituximab","Siponimod","Teriflunomide"))
xrange <- range(c(30,55))
yrange <- range(c(-30,80))

#creat grid structure
par(mfrow = c(3, 4))
par(cex = 0.6)
par(mar = c(0.5, 0.5, 0.5, 0.5), oma = c(4, 4, 0.5, 0.5))
par(tcl = -0.25)
par(mgp = c(2, 0.6, 0))

type<-unique(ddd2$Factors)

#iterate over factors to create individual bubble plots
for (i in 1:length(type)) {
```

```r
j<-which(ddd2$Factors==as.character(type[i]))

if(i==12){1}

else{
  if(i %in% c(1,5)){plot(xrange,yrange,xaxt='n',type='n',col.axis = "grey20", cex.axis=1.5)}
  if(i %in% 10:13){plot(xrange,yrange,yaxt='n',type='n',col.axis = "grey20", cex.axis=1.5)}
  if(i==9){plot(xrange,yrange,type='n', col.axis = "grey20", cex.axis=1.5)}
  if(i %in% c(2:4,6:8)){plot(xrange,yrange,axes=F,type='n', col.axis = "grey20", cex.axis=1.5)}

  par(new=T)

  #drug labels
  if(i==7){mtext("Laquinimod/Siponimod", side = 3, line = -1.5, cex = 1.1, col = "black")}
  else{mtext(unique(type.f)[i], side = 3, line = -1.5, cex = 1.1, col = "black")}

  box(col = "grey60")

  par(new=T)

  #plot laquinimod and siponimod together
  if(i==7){
    #draw bubbles for therapy using the fact that area = pi*r^2
    with(ddd2, symbols(x=Age[c(30:31,40)], y=DI[c(30:31,40)], circles=sqrt(Weight[c(30:31,40)]/pi),
                       inches=0.25*sqrt(max(Weight[c(30:31,40)])/max(ddd2$Weight)),
                       ann=F, fg="darkcyan", xlim = xrange, ylim=yrange,
                       xlab = "", ylab = "", xaxt='n',yaxt='n', pch=16,lwd=1.5))
  }

  else{
    #draw bubbles for therapy using the fact that area = pi*r^2
    with(ddd2, symbols(x=Age[j], y=DI[j], circles=sqrt(Weight[j]/pi),
                       inches=0.25*sqrt(max(Weight[j])/max(ddd2$Weight)),
                       ann=F, fg="darkcyan", xlim = xrange, ylim=yrange,
                       xlab = "", ylab = "", xaxt='n',yaxt='n', pch=16,lwd=1.5))
  }

  par(new=T)

  #create band for confidence interval
  polygon(c(rev(newx.df), newx.df), c(rev(unique(preds.ddd2[ ,3])), unique(preds.ddd2[,2])),
          col=rgb(0, 0, 0,0.05), border = NA)

  #add best fit line
  abline(coef(ddd2.both.fit),lty=2)
```

```
    #draw dashed lines for confidence intervals
    lines(newx.df, preds.ddd2[ ,3], lty = 'dashed', col = 'black')
    lines(newx.df, preds.ddd2[ ,2], lty = 'dashed', col = 'black')
  }
}

#label axes
mtext("Age (years)", side = 1, outer = TRUE, cex = 1.3, line = 2.6, col = "grey20")
mtext("Inhibition of Disability Progression (%)", side = 2, outer = TRUE, cex = 1.3, line = 2.3,col = "grey20")

dev.off()

#----------------------------------------------------------------
#Compute weighted standardized residuals (Figure 4 bar charts)
#----------------------------------------------------------------

wres.approve<-c() #vector for residuals of FDA-approved drugs (for approved MS subtypes)
wres.all<-c() #vector for all residuals

#----------------------------------------

#Alemtuzumab (all trials FDA-approved dose in RRMS)
wres.approve$A<-mean(weighted.residuals(ddd.approve.fit)[1:3]/(summary(ddd.approve.fit)$sigma))

#Daclizumab (all trials FDA-approved dose in RRMS)
wres.approve$D<-mean(weighted.residuals(ddd.approve.fit)[4:5]/(summary(ddd.approve.fit)$sigma))

#Dimethyl Fumarate (all trials FDA-approved dose in RRMS)
wres.approve$DF<-mean(weighted.residuals(ddd.approve.fit)[6:7]/(summary(ddd.approve.fit)$sigma))

#Fingolimod (3 trials FDA-approved dose in RRMS)
wres.approve$F<-mean(weighted.residuals(ddd.approve.fit)[8:10]/(summary(ddd.approve.fit)$sigma))

#Glatiramer acetate (5 trials FDA-approved dose in RRMS)
wres.approve$G<-mean(weighted.residuals(ddd.approve.fit)[11:15]/(summary(ddd.approve.fit)$sigma))

#Inteferon-beta (6 trials FDA-approved dose in RRMS)
wres.approve$I<-mean(weighted.residuals(ddd.approve.fit)[16:21]/(summary(ddd.approve.fit)$sigma))

#Mitoxantrone (1 trial FDA-approved dose in SPMS)
wres.approve$M<-mean(weighted.residuals(ddd.approve.fit)[22]/(summary(ddd.approve.fit)$sigma))

#Natalizumab (2 trials FDA-approved dose in RRMS)
wres.approve$N<-mean(weighted.residuals(ddd.approve.fit)[23:24]/(summary(ddd.approve.fit)$sigma))

#Ocrelizumab (all trials FDA-approved dose in RRMS and PPMS)
wres.approve$O<-mean(weighted.residuals(ddd.approve.fit)[25:27]/(summary(ddd.approve.fit)$sigma))
```

```
#Teriflunomide (all trials FDA-approved dose in RRMS)
wres.approve$T<-mean(weighted.residuals(ddd.approve.fit)[28:31]/(summary(ddd.approve.fit)$sigma))

wres.approve.labels<-c("Alemtuzumab","Daclizumab","Dimethyl Fumarate","Fingolimod","Glatiramer Acetate",
                       "Interferon Beta","Mitoxantrone","Natalizumab","Orelizumab","Teriflunomide")
wres.approve.total<-c(wres.approve$A,wres.approve$D,wres.approve$DF,wres.approve$F,wres.approve$G,wres.approve$I,
                      wres.approve$M,wres.approve$N,wres.approve$O,wres.approve$T)

#Color-coded lollipop plot of residuals for approved therapies
tiff("Lollipop_Approve.tiff", width = 18, height = 14, units = 'in', res = 300)

df.approve<-data.frame(x=wres.approve.total,y=wres.approve.labels,color=legend.approve)
df.approve$y<-factor(df.approve$y, levels=df.approve[order(df.approve$x,decreasing=F),]$y)
df.approve$stick<-sapply(df.approve$x,function(x)
  if(x>0.03){round(x,2)-0.095}
  else if(x<0){round(x,2)+0.095}
  else{0})
ggplot(df.approve, aes(x=df.approve$y,y=round(df.approve$x,2),label=round(df.approve$x,2))) +
  theme_bw()+
  geom_segment(aes(y = 0,
                   x = df.approve$y,
                   yend = df.approve$stick,
                   xend = df.approve$y),
               color = "black") +
  geom_point(size=32.3,shape=1,colour="black",show.legend=F)+
  geom_point(colour=df.approve$color,fill=df.approve$color,show.legend=F, size=32,alpha=0.5)  +
  geom_text(color="black",size=9) +
  labs(y="Mean of Standardized Weighted Residuals",x=NULL,title="FDA-Approved Drug Trials")+
  ylim(-1, 1.5) +
  theme(plot.title = element_text(size=36, face="bold"),
        axis.text.x=element_text(colour="black",size=25,margin = margin(t = 10, r = 10, b = 10, l = 10)),
        axis.text.y=element_text(colour="black",size=30,margin = margin(t = 10, r = 10, b = 10, l = 10)),
        axis.title=element_text(size=31))+
  coord_flip()
dev.off()

#-----------------------------------------------

#Alemtuzumab (all trials)
wres.all$A<-mean(weighted.residuals(ddd2.both.fit)[1:3]/(summary(ddd2.both.fit)$sigma))

#Daclizumab (all trials)
wres.all$D<-mean(weighted.residuals(ddd2.both.fit)[4:5]/(summary(ddd2.both.fit)$sigma))

#Dimethyl Fumarate (all trials)
wres.all$DF<-mean(weighted.residuals(ddd2.both.fit)[6:7]/(summary(ddd2.both.fit)$sigma))
```

```
#Fingolimod (all trials)
wres.all$F<-mean(weighted.residuals(ddd2.both.fit)[c(8:11)]/(summary(ddd2.both.fit)$sigma))

#Glatiramer acetate (all trials)
wres.all$G<-mean(weighted.residuals(ddd2.both.fit)[c(12:17)]/(summary(ddd2.both.fit)$sigma))

#Inteferon-beta (all trials)
wres.all$I<-mean(weighted.residuals(ddd2.both.fit)[c(18:29)]/(summary(ddd2.both.fit)$sigma))

#Laquinimod (all trials)
wres.all$L<-mean(weighted.residuals(ddd2.both.fit)[30:31]/(summary(ddd2.both.fit)$sigma))

#Mitoxantrone (all trials)
wres.all$M<-mean(weighted.residuals(ddd2.both.fit)[32]/(summary(ddd2.both.fit)$sigma))

#Natalizumab (all trials)
wres.all$N<-mean(weighted.residuals(ddd2.both.fit)[33:35]/(summary(ddd2.both.fit)$sigma))

#Ocrelizumab (all trials)
wres.all$O<-mean(weighted.residuals(ddd2.both.fit)[36:38]/(summary(ddd2.both.fit)$sigma))

#Rituximab (all trials)
wres.all$R<-mean(weighted.residuals(ddd2.both.fit)[39]/(summary(ddd2.both.fit)$sigma))

#Siponimod (all trials)
wres.all$S<-mean(weighted.residuals(ddd2.both.fit)[40]/(summary(ddd2.both.fit)$sigma))

#Teriflunomide (all trials)
wres.all$T<-mean(weighted.residuals(ddd2.both.fit)[41:44]/(summary(ddd2.both.fit)$sigma))

wres.all.labels<-c("Alemtuzumab","Daclizumab","Dimethyl Fumarate","Fingolimod","Glatiramer Acetate",
                    "Interferon Beta","Laquinimod","Mitoxantrone","Natalizumab","Orelizumab",
                   "Rituximab","Siponimod","Teriflunomide")
wres.all.total<-c(wres.all$A,wres.all$D,wres.all$DF,wres.all$F,wres.all$G,wres.all$I,wres.all$L,
                  wres.all$M,wres.all$N,wres.all$O,wres.all$R,wres.all$S,wres.all$T)

#Color-coded lollipop plot of residuals for all therapies
tiff("Lollipop_All.tiff", width = 18, height = 14, units = 'in', res = 300)

df.all<-data.frame(x=wres.all.total,y=wres.all.labels,color=legend.all)
df.all$y<-factor(df.all$y, levels=df.all[order(df.all$x,decreasing=F),]$y)
df.all$stick<-sapply(df.all$x,function(x)
  if(x>0.03){round(x,2)-0.095}
  else if(x<0){round(x,2)+0.095}
  else{0})
ggplot(df.all, aes(x=df.all$y,y=round(df.all$x,2),label=round(df.all$x,2))) +
```

```r
  theme_bw()+
  geom_segment(aes(y = 0,
                   x = df.all$y,
                   yend = df.all$stick,
                   xend = df.all$y),
               color = "black") +
  geom_point(size=32.3,shape=1,colour="black",show.legend=F)+
  geom_point(colour=df.all$color,fill=df.all$color,show.legend=F, size=32,alpha=0.5)  +
  geom_text(color="black",size=9) +
  labs(y="Mean of Standardized Weighted Residuals",x=NULL,title="All Drug Trials")+
  ylim(-1, 1.5) +
  theme(plot.title = element_text(size=36, face="bold"),
        axis.text.x=element_text(colour="black",size=25,margin = margin(t = 10, r = 10, b = 10, l = 10)),
        axis.text.y=element_text(colour="black",size=30,margin = margin(t = 10, r = 10, b = 10, l = 10)),
        axis.title=element_text(size=31))+
  coord_flip()
dev.off()

#----------------------------------------------------------------
#Fit model with interaction term
#----------------------------------------------------------------

#convert to factor
ddd.miss$Efficacy<-as.factor(ddd.miss$Efficacy)

#fit weighted regression with interaction term via step-down procedure
ddd.miss.fitv0<-lm(DI~Age*Efficacy*EDSS,data=ddd.miss,weights=Weight,na.action=na.exclude); summary(ddd.miss.fitv0)
ddd.miss.fitv1<-lm(DI~Age*Efficacy+Age*EDSS+EDSS*Efficacy,data=ddd.miss,weights=Weight,na.action=na.exclude);
summary(ddd.miss.fitv1)
ddd.miss.fitv2<-lm(DI~Age*Efficacy+EDSS*Efficacy,data=ddd.miss,weights=Weight,na.action=na.exclude);
summary(ddd.miss.fitv2)
ddd.miss.fitv3<-lm(DI~Age*Efficacy+EDSS,data=ddd.miss,weights=Weight,na.action=na.exclude); summary(ddd.miss.fitv3)
ddd.miss.fit<-lm(DI~Age*Efficacy,data=ddd.miss,weights=Weight,na.action=na.exclude)

#check model fit
summary(ddd.miss.fit)
confint(ddd.miss.fit)
par(mfrow=c(2,2))
plot(ddd.miss.fit)
par(mfrow=c(1,1))

#residuals versus fitted
plot(residuals(ddd.miss.fit)~ddd.miss$Age,col=ddd.miss$Efficacy,pch=16+as.numeric(ddd.miss$Efficacy))

#effect plot
plot(effect("Age*Efficacy",mod=ddd.miss.fit,xlevels=list(Efficacy=c(1,2))))
```

```r
#---------------------------------------------------------------------
#Bubble Plot for model with interaction term (Figure 4C)
#---------------------------------------------------------------------
#set for export
tiff("Plot_both_high_fit.tiff", width = 19, height = 25, units = 'in', res = 300)
par(mfrow = c(2, 1),
    oma = c(3, 4, 1, 1),
    mar = c(7, 7, 3, 2),
    mgp = c(5, 2.5, 1))

#create plot window for data
plot(xrange, yrange,type = 'n',ylab="Inhibition of disability progression (%)",
     xlab="",main="FDA-Approved Drug Trials",cex.axis=2,cex.lab=2.75,cex.main=3)

#legend for FDA-approved therapies (inset in graph)
legend("topright",
       c("A = Alemtuzumab","D = Daclizumab","DF = Dimethyl Fumarate","F = Fingolimod","G = Glatiramer Acetate",
         "I = Interferon Beta","M = Mitoxantrone", "N = Natalizumab","O = Ocrelizumab",
         "T = Teriflunomide"), fill=legend.approve, cex=2)

lines(newx.df, predict(ddd.miss.fit, data.frame(Age = newx.df,
                                                Efficacy = factor(rep(1, length(newx.df))))),lty=2)

lines(newx.df, predict(ddd.miss.fit, data.frame(Age = newx.df,
                                                Efficacy = factor(rep(2, length(newx.df))))),lty=2)

par(new=T)

#predict confidence intervals
preds.ddd.miss1 <- predict(ddd.miss.fit,data.frame(Age = newx.df,
                                                   Efficacy = factor(rep(1, length(newx.df)))),interval='confidence')
preds.ddd.miss2 <- predict(ddd.miss.fit,data.frame(Age = newx.df,
                                                   Efficacy = factor(rep(2, length(newx.df)))),interval='confidence')

#create band for confidence interval
polygon(c(rev(newx.df), newx.df), c(rev(unique(preds.ddd.miss1[ ,3])), unique(preds.ddd.miss1[,2])),
        col=rgb(0, 0, 0,0.05), border = NA)

polygon(c(rev(newx.df), newx.df), c(rev(unique(preds.ddd.miss2[ ,3])), unique(preds.ddd.miss2[,2])),
        col=rgb(0, 0, 0,0.05), border = NA)

#draw dashed lines for confidence intervals
lines(newx.df, preds.ddd.miss1[ ,3], lty = 'dashed', col = 'black')
lines(newx.df, preds.ddd.miss1[ ,2], lty = 'dashed', col = 'black')
lines(newx.df, preds.ddd.miss2[ ,3], lty = 'dashed', col = 'black')
lines(newx.df, preds.ddd.miss2[ ,2], lty = 'dashed', col = 'black')
```

```r
par(new=T)

with(ddd3,symbols(x=Age, y=DI, circles=sqrt(Weight/pi),
                  inches=1.3, ann=F,
                  bg=color.approve,xlim = xrange, ylim=yrange, xlab = "", ylab = "", xaxt='n',yaxt='n',lwd=1.5))

par(new=T)

#ID bubbles
text(ddd3$Age,ddd3$DI,dimnames(ddd3)[[1]],cex=1.7)

box()

#-------------------------------------------------------------------------------------------------------
#Bubble plot for trials of high efficacy therapies (Figure 4E)
#-------------------------------------------------------------------------------------------------------

#create graphing parameters
xrange <- range(c(30,55))
yrange <- range(c(-25,80))
newx.df <- seq(-100,100, length.out=500)
temp <- data.frame(newx.df)
dimnames(temp)[[2]][1] <- "Age"

ddd3.high.fit<-lm(DI~Age,data=ddd3.high,weights=Weight)

#predict 95% confidence intervals
preds.ddd3.high <- predict(ddd3.high.fit,newdata=temp,interval='confidence')

#create plot window for data
plot(xrange, yrange,type = 'n',ylab="Inhibition of disability progression (%)",
     xlab="Age (years)",main="High Efficacy Drug Trials",cex.axis=2,cex.lab=2.75,cex.main=3)

#legend for high efficacy therapies (inset in graph)
legend("topright",
       c("A = Alemtuzumab","D = Daclizumab","M = Mitoxantrone", "N = Natalizumab","O = Ocrelizumab"),
       fill=legend.high, cex=2)

#draw shaded region of confidence band
polygon(c(rev(newx.df), newx.df), c(rev(preds.ddd3.high[ ,3]), preds.ddd3.high[ ,2]), col=rgb(0, 0, 0,0.05), border =
NA)

#add best fit line
abline(coef(ddd3.high.fit),lty=2)

#draw dashed lines for confidence intervals
lines(newx.df, preds.ddd3.high[ ,3], lty = 'dashed', col = 'black')
```

```r
lines(newx.df, preds.ddd3.high[ ,2], lty = 'dashed', col = 'black')

par(new=T)

#draw high efficacy bubbles using the fact that area = pi*r^2
with(ddd3.high,symbols(x=Age, y=DI, circles=sqrt(Weight/pi),
                       inches=1.3, ann=F,bg=color.high,xlim = xrange, ylim=yrange, xlab = "",
                       ylab = "", xaxt='n',yaxt='n',lwd=1.5))

par(new=T)

#ID bubbles
text(ddd3.high$Age,ddd3.high$DI,dimnames(ddd3.high)[[1]],cex=1.7)

dev.off()

#------------------------------------------------------------------------------------
#Bubble plot for trials of low efficacy therapies (Figure 4D)
#------------------------------------------------------------------------------------

#set for export
tiff("Plot_low_ttest_fit.tiff", width = 19, height = 25, units = 'in', res = 300)
par(mfrow = c(2, 1),
    oma = c(3, 4, 1, 1),
    mar = c(7, 7, 3, 2),
    mgp = c(5, 2.5, 1))

#create graphing parameters
xrange <- range(c(30,55))
yrange <- range(c(-25,80))
newx.df <- seq(-100,100, length.out=500)
temp <- data.frame(newx.df)
dimnames(temp)[[2]][1] <- "Age"

#add best fit lines
ddd3.low.fit<-lm(DI~Age,data=ddd3.low,weights=Weight)

#predict 95% confidence intervals
preds.ddd3.low <- predict(ddd3.low.fit,newdata=temp,interval='confidence')

#create plot window for data
plot(xrange, yrange,type = 'n',ylab="Inhibition of disability progression (%)",
     xlab="",main="Low Efficacy Drug Trials",cex.axis=2,cex.lab=2.75,cex.main=3)

#legend for low efficacy therapies (inset in graph)
legend("topright", c("DF = Dimethyl Fumarate","F = Fingolimod","G = Glatiramer Acetate",
                     "I = Interferon Beta", "T = Teriflunomide"), fill=legend.low, cex=2)
```

```
#draw shaded region of confidence band
polygon(c(rev(newx.df), newx.df), c(rev(preds.ddd3.low[ ,3]), preds.ddd3.low[ ,2]), col=rgb(0, 0, 0,0.05), border = NA)

#add best fit line
abline(coef(ddd3.low.fit),lty=2)

#draw dashed lines for confidence intervals
lines(newx.df, preds.ddd3.low[ ,3], lty = 'dashed', col = 'black')
lines(newx.df, preds.ddd3.low[ ,2], lty = 'dashed', col = 'black')

par(new=T)

#draw low efficacy bubbles using the fact that area = pi*r^2
with(ddd3.low,symbols(x=Age, y=DI, circles=sqrt(Weight/pi),
                      inches=1.3*sqrt(max(Weight)/max(ddd2$Weight)), ann=F,
                      bg=color.low,xlim = xrange, ylim=yrange, xlab = "", ylab = "", xaxt='n',yaxt='n',lwd=1.5))

par(new=T)

#ID bubbles
text(ddd3.low$Age,ddd3.low$DI,dimnames(ddd3.low)[[1]],cex=1.7)

box()

#-------------------------------------------------------------------
#t-test for difference in means (Figure 4F)
#-------------------------------------------------------------------

#create graphing parameters
xrange <- range(c(30,55))
yrange <- range(c(-50,50))
newx.df <- seq(-100,100, length.out=500)
temp <- data.frame(x=newx.df)
dimnames(temp)[[2]][1] <- "Age"

#compute differences in means between high and low efficacy data
ddd.miss.fake<-data.frame(Age=rep(seq(from=25,to=60,length.out=100),1),Efficacy=factor(rep(c(2),each=100)))
newx<-rbind(model.matrix(ddd.miss.fit)[1:30,],model.matrix(ddd.miss.fit)[1:30,],
            model.matrix(ddd.miss.fit)[1:30,],model.matrix(ddd.miss.fit)[1:10,])
newx[,1:2]<-0
newx[,3]<-1
newx[,4]<-ddd.miss.fake$Age
SEs<-sqrt(diag(newx%*%vcov(ddd.miss.fit)%*%t(newx)))
estdiff<-newx%*%ddd.miss.fit$coefficients

plot(xrange, yrange,type = 'n',ylab="Difference in Means",
```

```
     xlab="Age (years)",main="Difference between High & Low Efficacy Drugs",cex.axis=2,cex.lab=2.75,cex.main=3)

#add best fit line to data
abline(lm(estdiff~ddd.miss.fake$Age),lty=1,lwd=2)

#compute 95% confidence interval estimates (difference in means, t-test)
SEs<-sqrt(diag(newx%*%vcov(ddd.miss.fit)%*%t(newx)))
lines(estdiff-qt(.975,df=33)*SEs~ddd.miss.fake$Age,lwd=2,lty=2,col="red")
lines(estdiff+qt(.975,df=33)*SEs~ddd.miss.fake$Age,lwd=2,lty=2,col="red")
abline(h=0,col="gray15",lwd=2)
abline(v=40.5,col="gray15",lwd=2,lty=2)

dev.off()
```
